# Phytosulfokine signalling blocks mycotoxin toxicity in Arabidopsis and mediates suppression of cell death activated by bacterial microbe‐associated molecular patterns

**DOI:** 10.1111/nph.70811

**Published:** 2025-12-12

**Authors:** Ali O. Alqarni, John M. U. Hamilton, Adrian P. Brown, Stephen Chivasa

**Affiliations:** ^1^ Department of Biosciences Durham University South Road Durham DH1 3LE UK; ^2^ Department of Biology, College of Arts and Sciences Najran University Najran 61442 Saudi Arabia

**Keywords:** *Arabidopsis thaliana*, BAK1, Calvin cycle, Fumonisin B1, microbe‐associated molecular pattern, phytosulfokine signalling, plant cell death, PSKR1

## Abstract

- Fumonisin B1 (FB1) is a mycotoxin that disrupts ceramide biosynthesis and kills plants. Prior activation with bacterial microbe‐associated molecular patterns (MAMPs), such as components of bacterial flagella, effectively suppresses FB1‐induced cell death. The molecular basis of this defence against mycotoxin toxicity is poorly understood.
- Analysis of extracellular peptide receptors provided initial circumstantial evidence linking phytosulfokine (PSK) signalling with *Arabidopsis thaliana* responses to FB1. We used synthetic PSK peptides and quantitative proteomics to investigate this link and established the basis for peptide‐induced Arabidopsis immunity to FB1.
- Exogenous PSK fully protected Arabidopsis plants from FB1 toxicity in wild‐type plants, but not in loss‐of‐function mutants lacking PSK RECEPTOR 1 (PSKR1) or its co‐receptor BRASSINOSTEROID INSENSITIVE 1‐ASSOCIATED RECEPTOR KINASE 1 (BAK1). Mutants lacking the precursor PSK‐processing subtilase (SBT3.8) enzyme were more sensitive to FB1. The partial flagellin peptide flg22, which activates innate immunity to block FB1 toxicity in wild‐type plants, failed to rescue *pskr1* mutants, indicating that PSK signalling functions downstream of flg22. Proteomic analysis revealed Calvin cycle downregulation by FB1, while co‐application of the toxin with PSK increased Calvin cycle capacity.
- Our study reveals that the mechanism of disabling mycotoxin toxicity by MAMPs is activation of PSK signalling and stimulation of the photosynthetic machinery.

## Introduction

Plant cells communicate with their neighbours to activate growth and developmental programmes. While cell–cell communication by signal transmission through plasmodesmata plays key roles in plant development, apoplast signalling has also emerged as a crucial mode of cell communication in growth and stress‐adaptive processes (Chivasa & Goodman, 2020). Many secreted peptides of pathogen or plant origin signal in the apoplast through plasma membrane receptor kinases as highlighted in a previous review (He *et al*., 2018). The cell‐proliferation‐inducing phytosulfokine (PSK) peptide signalling is initiated at the plasma membrane through activation of PSK receptor (PSKR) proteins (Matsubayashi *et al*., 2002), and the range of physiological processes regulated by PSK peptide is only beginning to emerge. During our research, we observed an unexpected link between PSK peptide signalling and Arabidopsis stress response to the fungal toxin fumonisin B1 (FB1).

PSK is a sulfated pentapeptide with the sequence Y(SO_3_H)‐I‐Y(SO_3_H)‐T‐G (Matsubayashi & Sakagami, 1996). In Arabidopsis, PSK is generated from 80 to 120 amino acid precursor polypeptides (Matsubayashi & Sakagami, 2006), which are processed by sulfation of specific tyrosine residues in the Golgi apparatus and proteolytic cleavage in the apoplast. Tyrosine sulfation in Arabidopsis is catalysed by the single‐copy gene tyrosyl‐protein sulfotransferase (TPST) enzyme (Komori *et al*., 2009). Proteolytic processing in the apoplast is by subtilisin‐like serine proteases, such as Arabidopsis AtSBT3.8 (Stührwohldt *et al*., 2021) and tomato SlPhyt2 (Reichardt *et al*., 2020). Sulfation is critical for bioactivity, as synthetic PSK peptides lacking this posttranslational modification are incapable of activating cell proliferation (Matsubayashi & Sakagami, 1996) or protoplast expansion (Ladwig *et al*., 2015) in cell suspension cultures or protoplasts, respectively.

PSKR was first identified in *Daucus carota* (Matsubayashi *et al*., 2002) and functionally conserved orthologues were subsequently identified in other species, such as rice (Yang *et al*., 2019) and Arabidopsis (Matsubayashi *et al*., 2006; Amano *et al*., 2007). PSK binds the extracellular domain of the leucine‐rich repeat receptor kinase (C. Wang *et al*., 2015), triggering recruitment of the co‐receptor BRASSINOSTEROID INSENSITIVE 1‐ASSOCIATED RECEPTOR KINASE 1 (BAK1) (Ladwig *et al*., 2015; J. Wang *et al*., 2015) and H^+^‐ATPase proteins AHA1 and AHA2 (Ladwig *et al*., 2015). The co‐receptor function of BAK1 in the PSKR–BAK1 heterodimer complex is indispensable for PSK signalling, as PSK fails to stimulate root elongation (Ladwig *et al*., 2015; J. Wang *et al*., 2015) and protoplast expansion (Ladwig *et al*., 2015) in *bak1* loss‐of‐function mutants. PSK signalling in the activation of protoplast expansion is also mediated by CYCLIC NUCLEOTIDE‐GATED CHANNEL 17 (CNGC17), as reflected by inhibition of this response in Arabidopsis *cngc17* loss‐of‐function mutants (Ladwig *et al*., 2015). Full details of signalling events proximate to receptor activation by PSK or the downstream molecular targets underpinning the physiological processes regulated by the peptide are still unclear.

PSK signalling regulates growth processes, such as stimulation of cell proliferation (Matsubayashi & Sakagami, 1996) and expansion (Stührwohldt *et al*., 2011), enhancing pollen germination (Chen *et al*., 2000), and pollen tube growth (Stührwohldt *et al*., 2015), regulation of hypocotyl (Stührwohldt *et al*., 2011) and root (Matsubayashi & Sakagami, 2006; Kutschmar *et al*., 2009) growth, and root nodulation (J. Wang *et al*., 2015). Roles for PSK in biotic and abiotic stress‐adaptive responses have also been reported. Impairment of proteolytic processing of precursor PSK polypeptides in Arabidopsis *sbt3.8* mutants results in severe growth impairment under osmotic stress relative to wild‐type, but this is alleviated by exogenous PSK (Stührwohldt *et al*., 2021). Heterologous expression of the rice OsPSKR15 protein in Arabidopsis enhances sensitivity to the drought stress‐protective hormone abscisic acid (ABA) and significantly improves growth performance under drought (Nagar *et al*., 2022). OsPSKR15 directly binds *Arabidopsis thaliana* PYRABACTIN RESISTANCE‐LIKE 9 (AtPYL9) and *Oryza sativa* OsPYL11 (Nagar *et al*., 2022) ABA receptors. Perhaps, this is the basis for PSK function in drought adaptation; however, the full mechanism is yet to be established.

PSK is a key regulatory signal that switches off immune responses for resumption of growth after pathogen attack. Thus, it has been proposed that exogenous PSK suppresses activation of pattern‐triggered immunity, while Arabidopsis *pskr1* mutants show enhanced resistance to biotrophic bacterial pathogens (Igarashi *et al*., 2012). By contrast, disruption of PSK signalling leads to increased susceptibility to necrotrophic fungi as reported for Arabidopsis *pskr1* mutants infected with *Alternaria brassicicola* (Mosher & Kemmerling, 2013). In tomato, exogenous PSK application suppressed infection by the necrotrophic *Botrytis cineria* pathogen, while silencing of *Solanum lycopersici* SlPSKR1 increased the infection (H. Zhang *et al*., 2018). As necrotrophic fungi secrete mycotoxins to weaken plant immune responses (Mosher & Kemmerling, 2013), these results may imply a role for PSK in the attenuation of mycotoxin action.

Our group has a long‐standing interest in understanding how plants respond to the cell death‐activating *Fusarium verticillioides* mycotoxin, fumonisin B1 (FB1). The Arabidopsis–FB1 interaction has gained widespread use as an experimental system to investigate the regulation of programmed cell death in plants (Asai *et al*., 2000; Stone *et al*., 2000; Chivasa *et al*., 2005; Shi *et al*., 2007; Kim *et al*., 2011). In this study, we show that exogenous PSK blocks FB1‐induced cell death. We used PSK to design a proteomic experiment for identifying the cellular targets of PSK signalling in suppression of cell death. We report that photosynthesis is a key target for PSK signalling and that growth‐promoting peptide hormones counteract stress by targeting the Calvin cycle.

## Materials and Methods

### Plant and biological materials

Growth conditions described previously (Chivasa *et al*., 2005) were used for cultivating *Arabidopsis thaliana* L. plants in soil or tissue culture. T‐DNA insertion mutants and wild‐type plants were in the Columbia‐0 (Col‐0) ecotype. All mutant lines were purchased from the Nottingham Arabidopsis Stock Centre (Nottingham, UK). From the Syngenta Arabidopsis Insertion Library (Sessions *et al*., 2002), we obtained the T‐DNA insertion lines *pskr1‐6* (SAIL_245_H03) previously confirmed by Rodiuc *et al*. (2016) and the *pskr1‐2* line (SAIL_673_H07) previously confirmed by Matsubayashi & Sakagami (2006); Amano *et al*. (2007). We used loss‐of‐function mutants from the SALK collection (Alonso *et al*., 2003): *bak1‐4* (SALK_116202) and *bak1‐3* (SALK_034523), which were previously confirmed by Chinchilla *et al*. (2007), and *sbt3.8–1* (SALK_142870C) and *sbt3.8–2* (SALK_052039C). Fumonisin B1 (FB1) and alternariol were purchased from Santa Cruz Biotechnology (Santa Cruz, TX, USA) and Merck (Dorset, UK), respectively. Arabidopsis ecotype Wassilewskija‐0 (Ws‐0) line transformed with the wildtype *FLAGELLIN SENSITIVE 2* (*FLS2*) gene of Col‐0 fused to the gene encoding the GREEN FLUORESCENT PROTEIN was generated and characterised by Robatzek *et al*. (2006). PSK‐α (YIYTQ) and flg22 peptides were synthesised by Biosynth Laboratories (Billingham, UK), while PSY1 and RGF7 peptides were synthesised by SciLight Biotechnology Ltd (Beijing, China).

### Genotyping knockout mutants

Phire Plant Direct PCR kits (Thermo Scientific, MA, USA) were used for genotyping Arabidopsis T‐DNA insertion knockout lines following the manufacturer's instructions. Briefly, *c*. 5‐mm‐diameter leaf discs were cored and homogenised in 20 μl of sample buffer. The insoluble plant material was removed by centrifugation (10 000 ***g***, 10 min), and the DNA‐containing supernatant was diluted fivefold. Aliquots of 5 μl diluted sample were used as a DNA template for PCR using relevant primers. DNA from wild‐type Col‐0 plants was used as a negative control. The following primers were used: *PSKR1* (At2g02220) 5′‐GTTGCTCATTTAAATTGCTTGAGAC‐3′ and 5′‐CCACTACTTGCTGTTGCGTG‐3′, *BAK1* (At4g33430) 5′‐TCGGCGTTCCTTCTAATCGG‐3′ and 5′‐AATGATCCAGCCAGACACGG‐3′, SBT3.8 (At4g10540) 5′‐TACTTTCGTTGCACATGCCG‐3′ and 5′‐ACAGAAGCGTCTCTGGTGTC‐3′. *FLCY* (At5g63910) served as a reference control gene and was PCR‐amplified with the primer pair: 5′‐TCCCGATAGCAATCTCCCTTC‐3′ and 5′‐ACGACAAATCTCTTCCCATCCC‐3′.

### Treatment of plants

Soil‐grown plants were mock‐treated or treated with 5 μM FB1 ± 100 nM PSK by infiltration of three leaves per plant using a syringe without a needle. Where concentrations differing from these were used, they are indicated in the figure legends of the results. For RNA extraction, three biological replicate samples were generated by pooling leaves from three independent plants (one leaf from each) within each treatment per timepoint. For qualitative cell death assessments, at least six replicate plants were treated, and representative leaves were excised 7 d after treatment for photographing.

Tissue culture plant treatments were performed by sowing surface‐sterilised wild‐type Col‐0 seed on Murashige & Skoog basal medium agar plates (Chivasa *et al*., 2005) containing 100 nM FB1 ± 100 nM PSK, PSY1, or RGF7. After 3 d of stratification in darkness at 4°C, the plants were transferred to a growth chamber set at 22°C with a 16‐h photoperiod (120 μmol m^−2^ s^−1^). At 15 d after transfer to the light–dark cycle, the Arabidopsis plants were harvested for protein extraction. Treatments contained four biological replicates, each consisting of a pool of *c*. 20 plants.

### Quantitative phytotoxicity symptoms assay

Leaf Chl content was used as a proxy to quantitatively assay phytotoxic symptoms and cell death in soil‐grown Arabidopsis plants treated with fumonisin B1 and alternariol. Three leaves per plant were infiltrated with 1–10 μM mycotoxin (FB1 or AOH) solutions (specific concentrations are provided in the figure legends of respective results). Three biological replicates, each consisting of leaves pooled from three independent plants, were generated at 3, 5, and/or 7 d after treatment and stored at −80°C. The tissues were homogenised in liquid nitrogen with a mortar and pestle. The powder was weighed and transferred into 1.5‐ml amber microfuge tubes containing 1 ml of 80% acetone. After vortex‐mixing and centrifugation (10 000 ***g***, 10 min), the supernatants were transferred into cuvettes for spectrophotometry. Chl*a* and Chl*b* were measured against an 80% acetone blank at wavelengths 663 nm and 645 nm, respectively. Chlorophyll concentration was calculated using the formula: Total Chl (*a* + *b*) = (20.2 × *A*_645_ + 8.02 × *A*_663_) × V/W, where: *A*_663_ = absorbance at 663 nm (Chl*a*), *A*_645_ = absorbance at 645 nm (Chl*b*), *V* = volume of extraction solvent (1 ml), and *W* = weight of leaf tissue (mg) (Arnon, 1949).

### RNA extraction and gene expression analyses

RNA extraction was performed using the Spectrum Plant Total RNA Kit (Merck, Dorset, UK), and cDNA synthesis was performed using the qPCRBIO cDNA Synthesis Kit (PCR Biosystems, London, UK) according to the manufacturer's instructions. Quantitative real‐time polymerase chain reaction and data analysis were conducted as previously reported (Ngara *et al*., 2018) using *EIF4A* (At3g13920) and *ACTIN2* (At3g18780) as constitutive reference controls. The following primer pairs were used: *PSKR1* (At2g02220) 5′‐ACCTTCCGGTGGTCAGTTT‐3′ and 5′‐AATCTGTGTTCCCCGCAGAG‐3′, *BAK1* (AT4G33430) 5′‐AGGTGTTCTCTTGGAGACTAGG‐3′ and 5′‐AGGTGTTCTCTTGGAGACTAGG‐3′, *PSYR1* (At1g17230) 5′‐CTGCAATTTCCGAACTTTTTGC‐3′ and 5′‐TTTGCTGACTTCAAGGTGATGC‐3′, *PSYR2* (At2g33170), 5′‐AGAGCAAGGCGGAGATCTTG‐3′ and 5′‐AGCACGGCAATTTTCGTCAC‐3′, *PSYR3* (At5g63930) 5′‐GGGAGAGGAGCTTGTGGAAC‐3′ and 5′‐CGCCTTCATGGTTTGATGCC‐3′, *RGI4* (AT5G56040) 5′‐GCCGGTAAAGGAAGTGGAGG‐3′ and 5′‐ATCGAAGCATGTTCTGGAGCC‐3′, *RBCL* (AtCg00490) 5′‐GGAGGAACTTTAGGCCACCC‐3′ and 5′‐TCGACTGCAAGATCACGTCC‐3′, *RBCS1A* (At1g67090) 5′‐GCACCACTCCACCATCACAC‐3′ and 5′‐CGGTACACAAATCCGTGCTC‐3′, and *RBCS3B* (At5g38410) 5′‐AGGATGGTCCACTTGAAAGG‐3′ and 5′‐TGGACTCTTATCCGCAAGCC‐3′. ANOVA and Tukey's *post hoc* tests were used for statistical data analysis.

### Protein extraction, iTRAQ analysis, and bioinformatics

A total of 12 samples from tissue culture plants consisting of three treatments (control, 100 nM FB1, and 100 nM FB1 + 100 nM PSK) and four biological replicates for each treatment were generated. Plant tissues were homogenised in liquid nitrogen with protein extraction buffer (10 mM Tris, 1 mM EDTA, 1% Triton X‐100, 1% 2‐mercaptoethanol, pH 8.0). The homogenates were centrifuged (20 000 *g*, 10 min) and the supernatant recovered for protein precipitation by the addition of 4 times the volume of 100% acetone and incubating for 12 h at −20°C. The precipitate was pelleted by centrifugation (10 000 **g**, 10 min) and washed twice with ice‐cold 80% acetone. The pellets were dissolved in a solution containing 9 M urea, 2 M thiourea, 4% CHAPS and protein concentration determined using a previously described method (Bradford, 1976).

Aliquots containing 20 μg protein were acetone‐precipitated and dissolved in 50 μl of 50 mM triethylammonium bicarbonate buffer (pH 8.5) containing 0.1% SDS. The proteins were reduced by incubation at 60°C for 1 h with 5 mM tris(2‐carboxyethylphosphine), followed by alkylation with 20 mM methyl‐methane‐thiol‐sulfonate in a 10‐min incubation at 20°C. The proteins were digested overnight using 2 μg trypsin at 37°C. Peptide labelling utilised an 8‐plex iTRAQ reagent kit (AB Sciex, Redwood City, CA, USA) following the manufacturer's instructions. The 4‐replicate control samples were labelled with 113, 114, 115, and 116 iTRAQ tags. Two sets of FB1‐treated samples were labelled: the first set with 113, 114, 115, and 116 iTRAQ tags, while the second set was labelled with 117, 118, 119, and 121 iTRAQ tags. The FB1 + PSK‐treated replicates were labelled with 117, 118, 119, and 121 iTRAQ tags. Two sets of pooled samples were generated as follows: controls pooled with FB1 samples (mixing 113, 114, 115, 116, 117, 118, 119 and 121) and FB1 pooled with FB1 + PSK samples (mixing 113, 114, 115, 116, 117, 118, 119 and 121). The two composite samples were then freeze‐dried and resuspended in 3% acetonitrile, 0.1% formic acid. The peptide samples were cleaned using hydrophilic interaction liquid chromatography (HILIC) SPE cartridges (PolyLC Inc., Columbia, MD, USA) according to a previously published protocol (Goche *et al*., 2020). For liquid chromatography–tandem mass spectrometry (LC‐MS/MS) analysis, samples were injected into an Eksigent 425 LC system through a Sciex Nanospray III source (AB Sciex), linked to a Triple TOF 6600 mass spectrometer (AB Sciex, Redwood City, CA, USA). Mass spectrometry data acquisition used the Analyst TF 1.7.1 software for instrument control and data processing (AB Sciex).

proteinpilot 5.0.1 revision 4895, which integrates the Paragon Algorithm 5.0.1.0.4874 (AB Sciex), was used to process the raw.wiff data files against an in‐house Arabidopsis database containing 27 380 protein sequences (downloaded February 2017) plus 244 non‐Arabidopsis contaminants. Search parameters and workflow used were as previously published (Smith *et al*., 2015). The data were manually filtered to exclude proteins identified based on less than 2 peptides sequenced at ≥ 95% confidence level. The second filter was applied to the control vs FB1 dataset to retain proteins whose response to FB1 was statistically significant (*P* ≤ 0.05). The third filter applied the *P* ≤ 0.05 significance threshold to the FB1 vs FB1 + PSK dataset. The 2 filtered protein sets were combined to give a final list of differentially expressed proteins, which were submitted for Gene Ontology and enrichment analysis using shinygo 0.77 (Ge *et al*., 2020) an online tool (http://bioinformatics.sdstate.edu/go) maintained by South Dakota State University.

## Results

### PSKR1 is upregulated in response to FB1 treatment

**Fig. 1:**
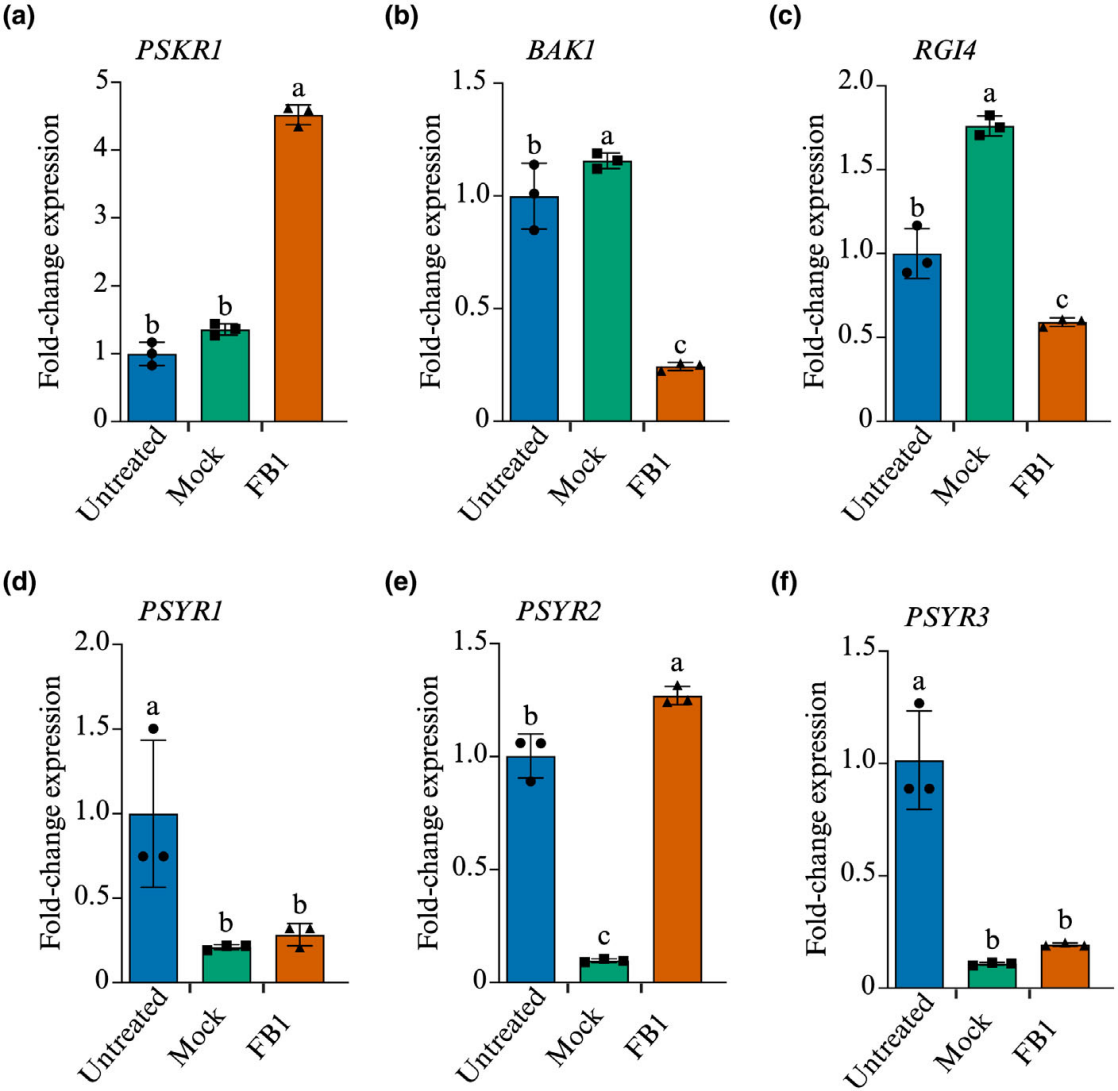
FB1 activates *PSKR1* and *BAK1* expression. *Arabidopsis thaliana* ecotype Col‐0 plants were infiltrated with 5 μM FB1 or mock‐treated with carrier solution. Leaf samples were harvested at 48 h for RNA extraction and qRT‐PCR analysis using primers designed to amplify (a) *PSKR1*, (b) *BAK1*, (c) *RGI4*, (d) *PSYR1*, (e) *PSYR2*, and (f) *PSYR3*. Bars represent mean ± SD (*n* = 3). Statistical analysis was performed using one‐way ANOVA and Tukey's test. Bars that do not share the same letter are significantly different (*P* ≤ 0.05). FB1, Fumonisin B1; Col‐0, Columbia‐0.

We have a long‐standing interest in extracellular signals governing plant stress‐adaptive responses (Chivasa & Goodman, 2020), and we currently focus on sulfated peptide signalling through their cognate plasma membrane receptors (reviewed in He *et al*., 2024). Thus, we investigated the response to FB1 treatment of genes encoding known sulfated peptide receptors PSKR1, PLANT PEPTIDE‐CONTAINING SULFATED TYROSINES RECEPTOR 1 (PSYR1), PSYR2, PSYR3, and ROOT MERISTEM GROWTH FACTOR 1‐INSENSITIVE 4 (RGI4), together with their shared co‐receptor BAK1. Samples for RNA extraction were harvested 48 h after treatment, a timepoint at which irreversible commitment to cell death occurs (Chivasa *et al*., 2005) and peak suppression of the gene encoding the pro‐cell death protein PLCL1 occurs in the FB1‐resistant *sid2* mutant (Smith *et al*., 2021). Despite these molecular changes, leaves treated with FB1 remain symptomless at 48 h, with the first signs of cell death appearing 24 h later at 72 h. *PSKR1* was significantly upregulated (> 5 fold) in response to 5 μM FB1 (Fig. 1a), while the common co‐receptor *BAK1* and *RGI4* were suppressed (Fig. 1b,c). There was a mixed picture with the PSY1 receptors (Fig. 1d–f). *PSYR1 and PSYR3* were suppressed by the mock treatment and did not at all respond to FB1 treatment, while there was a very modest increase (< 1.5 fold) in *PSYR2* expression. Since the shared co‐receptor gene *BAK1* was massively suppressed, it implies restricted capacity for signal flux through all the peptide receptors. However, we cannot confirm that these gene expression changes are reflected at the protein level; hence, any conclusions require validation by western blotting experiments or confirmation using gene knockout mutants. Notwithstanding the extrapolated suppression of BAK1 protein expression, the activation of *PSKR1* expression may reflect a ‘failed attempt’ to block FB1‐induced cell death through increased PSK signalling.

### Phytosulfokine signalling blocks FB1‐induced cell death

**Fig. 2:**
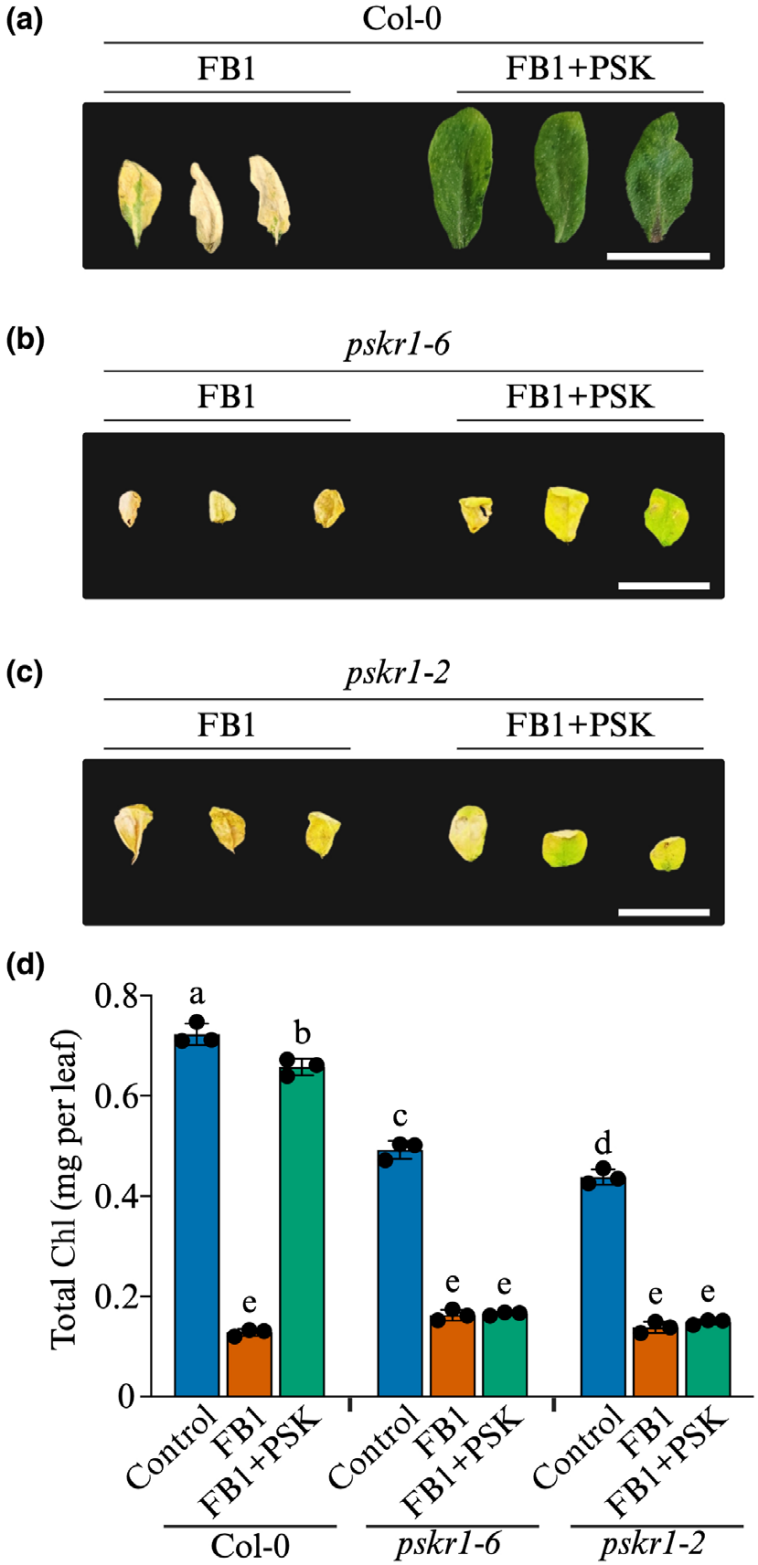
Arabidopsis PSKR1 is essential for cell death inhibition by exogenous PSK. Representative leaf images of (a) wild‐type Col‐0, (b) *pskr1‐6*, and (c) *pskr1‐2* plants treated with 5 μM FB1 ± 100 nM PSK for 7 d. Bars, 20 mm. (d) Chl content measurements in leaves of the indicated genotypes 7 d after exposure to the indicated treatments. Bars represent mean ± SD (*n* = 3). Statistical analysis was performed using 2‐way ANOVA. Bars without the same letters are significantly different (*P* ≤ 0.05). PSKR1, PSK RECEPTOR 1; PSK, phytosulfokine; FB1, Fumonisin B1.

Since PSK is a cell proliferation signal, we wondered whether increased *PSKR1* expression seen in preceding results reflects a ‘failed attempt’ to block FB1 toxicity via enhanced PSK signalling. Peptide signalling can be enhanced by increasing the receptor concentration, peptide concentration, or both. We opted to increase PSK signalling by raising peptide levels concurrently with FB1 application. Therefore, we treated leaves of soil‐grown Arabidopsis Col‐0 plants with 5 μM FB1 or a mixture of 5 μM FB1 + 100 nM PSK. Seven days after exposure to FB1, treated leaves had severely bleached and were dead, while exogenous PSK prevented death (Fig. 2a) and even promoted leaf expansion over the 7‐d period (Supporting Information Fig. S1).

For quantitative analysis, we have previously utilised an electrolyte leakage conductivity assay as a proxy for cell death (Chivasa *et al*., 2013). While this method is a reasonable proxy for cell damage, it fails to fully capture significant chlorosis that occurs without necessarily triggering electrolyte leakage. More importantly, the conductivity assay is an *in vitro* system that uses detached leaf discs responding to the imposed treatment while undergoing senescence due to detachment from the plant. Since chlorosis is a distinctive hallmark of FB1 toxicity in Arabidopsis, we considered the use of Chl assays. Whereas electrolyte leakage focuses on the terminal cell dismantling stages, the Chl assay comprehensively integrates the biochemical responses from early‐stage chlorosis to terminal pigment destruction as the cells disintegrate.

Therefore, we switched to Chl assays to quantify the protective effects of PSK signalling on the Arabidopsis response to mycotoxin poisoning. Leaves of Col‐0 plants treated with 5 μM FB1, with extensive cell death, had a drastic reduction in Chl content (Fig. 2a,d). Co‐application of FB1 with 100 nM PSK prevented cell death and Chl destruction as reflected by the healthy appearance of the treated leaves (Fig. 2a,d). These results demonstrate that PSK signalling actively blocks FB1‐induced cell death, ascribing a new stress‐adaptive function to the PSK peptide growth hormone in Arabidopsis.

We used *pskr1* loss‐of‐function mutants (Fig. S2) to confirm the genetic basis for exogenous PSK function in blocking FB1‐induced cell death. The T‐DNA insertion mutant *pskr1‐2* was validated by Matsubayashi *et al*. (2006) and Amano *et al*. (2007), while *pskr1‐6* was validated by Rodiuc *et al*. (2016). Leaves of soil‐grown plants were infiltrated with 5 μM FB1 ± 100 nM PSK and harvested for photography and Chl assays 7 d later. While PSK blocked FB1‐induced cell death in wild‐type plants (Fig. 2a,d), it failed to rescue *pskr1* mutants from cell death, as indicated by Chl degradation and terminal cell death symptoms (Fig. 2b–d). This provides genetic evidence supporting a role for PSK signalling through the PSKR1 receptor in modulating mycotoxin responses. This supports the notion that PSK signalling may be recruited to operate a molecular switch that controls cell death in plants.

**Fig. 3:**
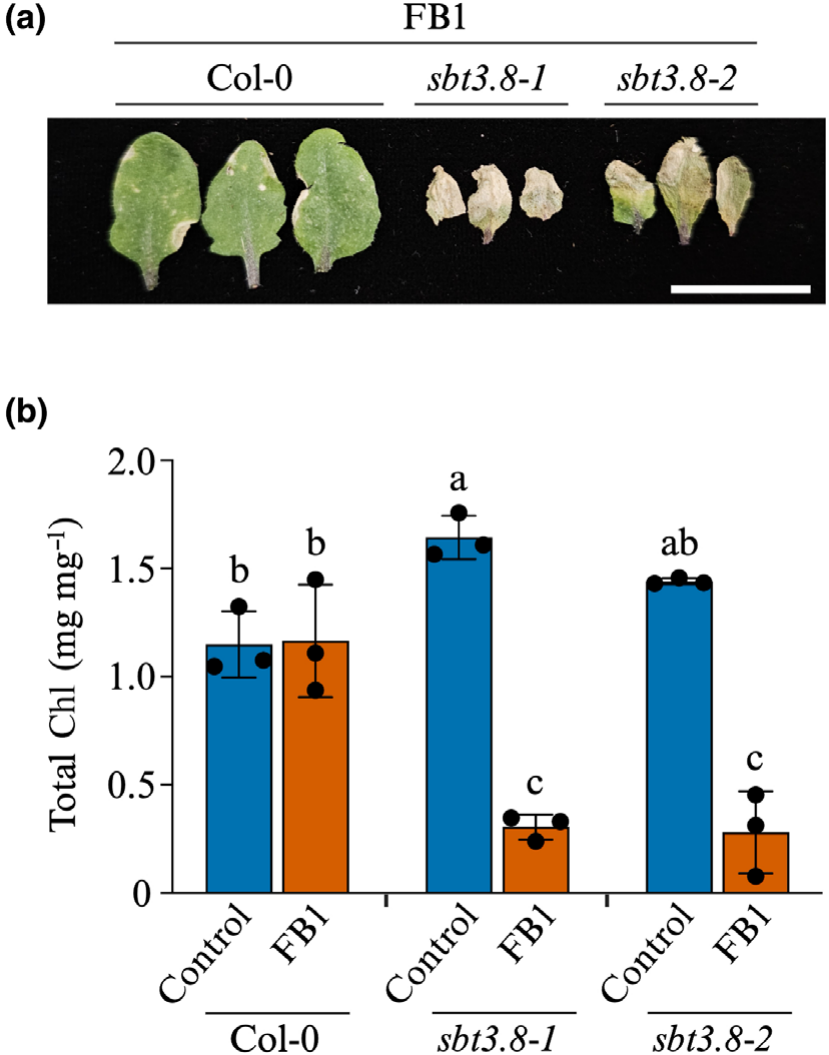
Processing of precursor PSK peptides is essential for PSK function. Arabidopsis wild‐type and *sbt3.8* mutant plants were infiltrated with 2.5 μM FB1. (a) Appearance of treated leaves 7 d after treatment. Bar, 20 mm. (b) Chl content in control and 2.5 μM FB1‐treated leaves at 7 d after treatment. Bars represent mean ± SD (*n* = 3). Statistical analysis was performed using 2‐way ANOVA. Bars without the same letters are significantly different (*P* ≤ 0.05). FB1, Fumonisin B1; PSK, phytosulfokine.

While the use of exogenous PSK clearly displayed cell death‐inhibitory properties in Arabidopsis, we wondered whether the converse would be true. Thus, we sought loss‐of‐function *sbt3.8* mutants (Fig. S2) lacking the functional subtilase required to process long precursor PSK peptides to the short bioactive pentapeptide used in PSK signalling. Leaves of wild‐type Col‐0 plants and *sbt3.8‐1* or *sbt3.8‐2* mutants were infiltrated with 2.5 μM FB1, a mycotoxin concentration that caused very minimal damage (if any) in the wild‐type plants. As expected, there was hardly any damage to Col‐0 plants 7 d after treatment as reflected by the healthy appearance of treated leaves (Fig. 3a) and no reduction in leaf Chl content (Fig. 3b). Leaves of both *sbt3.8‐1* and *sbt3.8‐2* mutants displayed severe damage at this low FB1 concentration (Fig. 3a) and the quantitative assay revealed massive destruction of Chl in the presence of the mycotoxin. Since these plants are expected to have reduced or no mature PSK (Stührwohldt *et al*., 2021), the high sensitivity of *sbt3.8* mutants lends further support to the notion that PSK regulates Arabidopsis' response to FB1.

### Exogenous PSK requires BAK1 to block FB1‐induced cell death

**Fig. 4:**
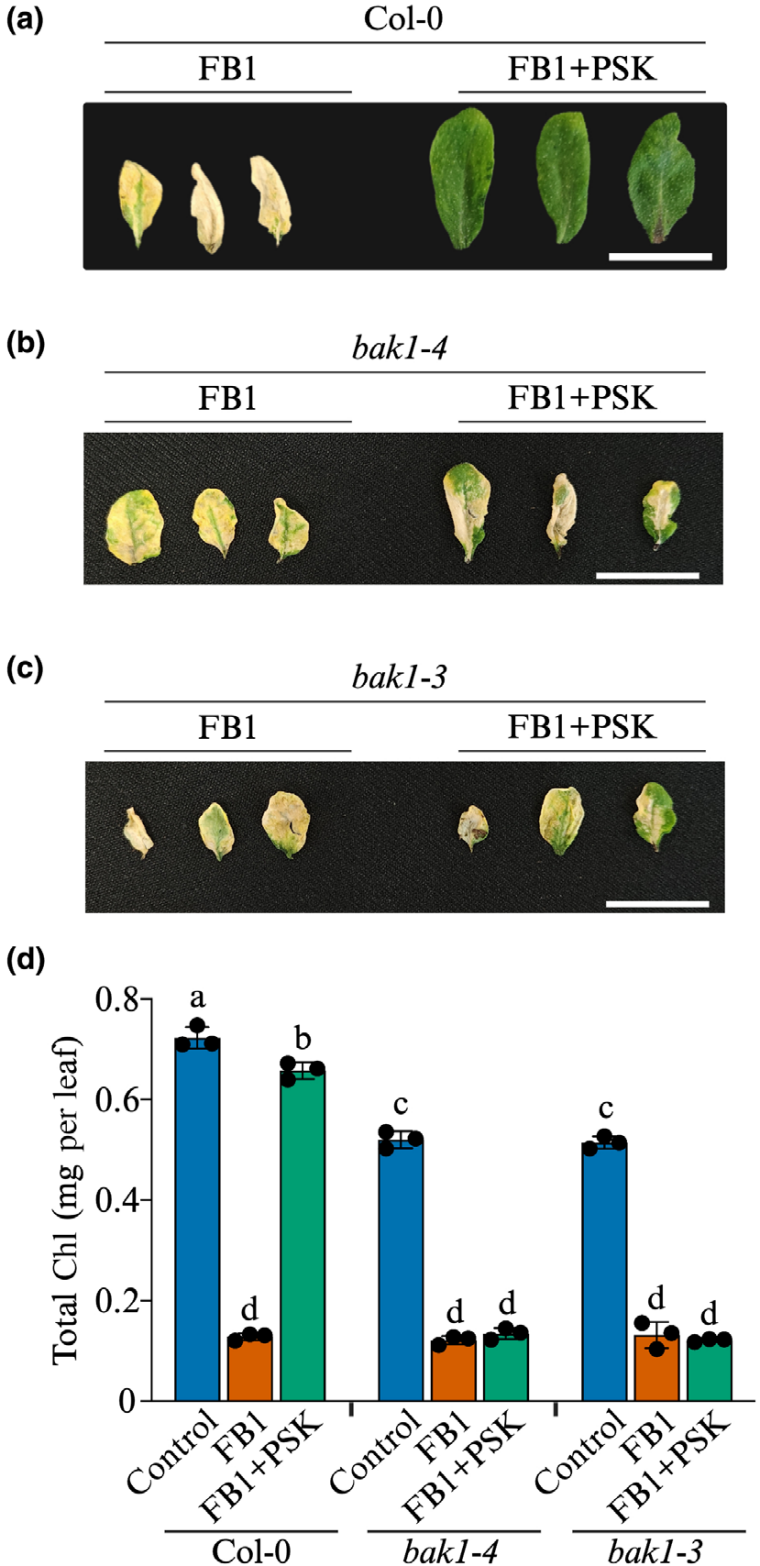
BAK1 is essential for PSK to block FB1 toxicity. (a–c) Arabidopsis soil‐grown plants of the indicated genotypes were treated with 5 μM FB1 ± 100 nM PSK, and photographs of treated leaves were taken 7 d later. Bar, 20 mm. (d) Chl content measured in FB1‐treated leaves at the 7 d timepoint. Bars represent mean ± SD (*n* = 3). Statistical analysis was performed using 2‐way ANOVA. Bars without the letters are significantly different (*P* ≤ 0.05). BAK1, BRASSINOSTEROID INSENSITIVE 1‐ASSOCIATED RECEPTOR KINASE 1; FB1, Fumonisin B1; PSK, phytosulfokine.

To understand the nature of signalling underpinning PSK‐mediated cell protection from FB1 toxicity, we investigated the potential role for BAK1, a known coreceptor that dimerises with PSKR1 (Ladwig *et al*., 2015; J. Wang *et al*., 2015) to form a functional BAK1–PSKR1 receptor complex essential for PSK signalling in root growth and protoplast expansion (Ladwig *et al*., 2015). To determine whether BAK1 is essential for PSK signalling in blocking cell death, we obtained loss‐of‐function *bak1‐3* and *bak1‐4* mutants (Fig. S2). Leaves of soil‐grown wild‐type and knockout mutant plants were infiltrated with 5 μM FB1 ± 100 nM PSK, and images of representative leaves were captured 7 d after treatment. While there were no noticeable differences between FB1‐treated wild‐type and mutant plants, the ability of PSK to block cell death was diminished in *bak1* mutants (Fig. 4a–c). Quantitative analysis of Chl content revealed a decline in *bak1* mutants treated with FB1, regardless of the presence or absence of PSK (Fig. 4d). By contrast, PSK preserved Chl levels in wild‐type plants (Fig. 4d). These results indicate that BAK1 is essential for PSK‐mediated signalling in blocking FB1‐induced cell death. Considered together with the results in Fig. 2, these findings clearly demonstrate the requirement for the PSKR1–BAK1 receptor complex in PSK modulation of cell death.

### Specificity of PSK in blocking FB1‐induced cell death

**Fig. 5:**
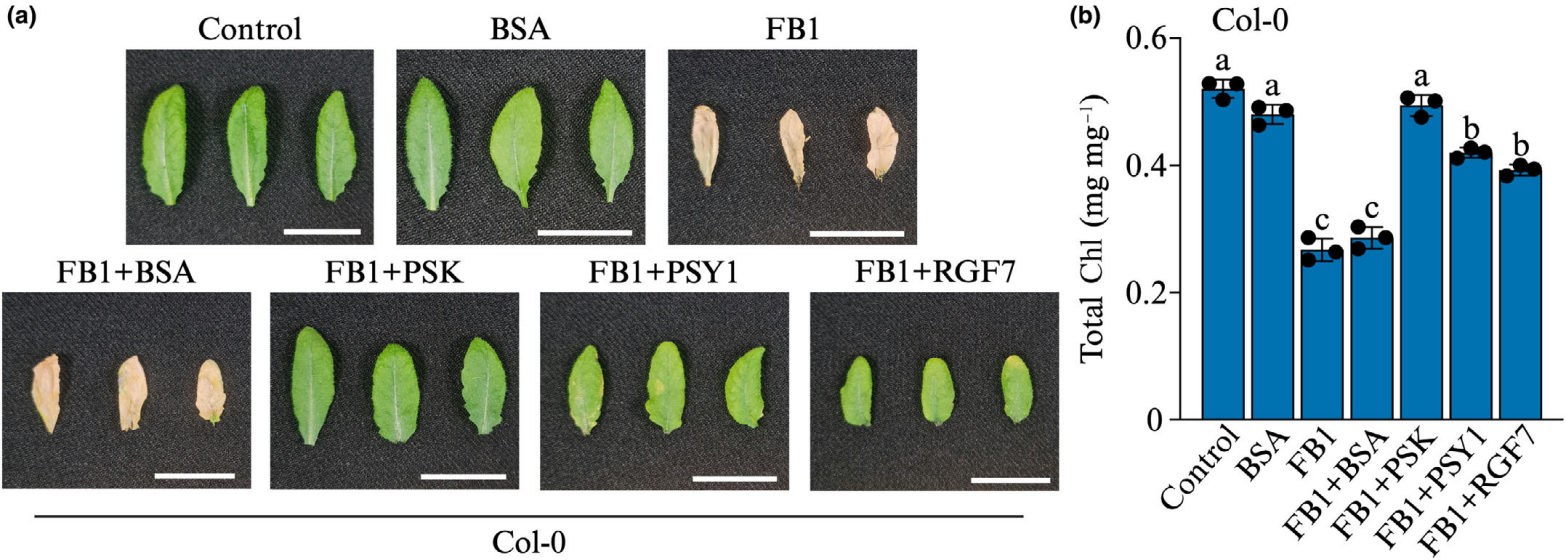
Effects of growth‐promoting peptide hormones on FB1 toxicity. (a) Representative images of Arabidopsis leaves from wild‐type Col‐0 plants treated with 5 μM FB1 ± 100 nM PSK, PSY1, or RGF7. Trypsin‐generated peptide mixture of bovine serum albumin (BSA) was included as a negative control. Photographs of representative leaves were taken 7 d post‐treatment. Bars, 20 mm. (b) Chl content was measured in leaves from Col‐0 plants at 7 d post‐treatment. Bars represent mean ± SD (*n* = 3). Statistical analysis was performed using one‐way ANOVA. Bars not sharing the same letters are significantly different (*P* ≤ 0.05). FB1, Fumonisin B1; PSK, phytosulfokine; Col‐0, Columbia‐0.

To test the specificity of PSK in protecting against FB1‐induced cell death, two additional sulfated growth‐promoting peptides, PSY1 and RGF7, were tested. Wild‐type Col‐0 plant leaves were infiltrated with 5 μM FB1 ± 100 nM PSK, PSY1, or RGF7. After 7 d, leaves treated with FB1 were completely dead as expected, while co‐treatment with PSK, PSY1, or RGF7 peptides blocked cell death (Fig. 5a). Quantitative Chl assays indicated that PSK offered the highest protection from FB1 toxicity, while PSY1 and RGF7 conferred slightly lower but highly significant defence against FB1 (Fig. 5b). A negative control peptide mixture generated by trypsin digestion of Bovine serum albumin (BSA) was included to investigate if the protection against FB1 was a non‐specific response to peptide infiltration into the apoplast or very specific to peptide growth hormones. Whereas PSK, PSY1, and RGF7 were able to block FB1‐induced cell death, BSA peptides failed to protect the leaf tissue from FB1 toxicity as indicated by the leaf images and Chl content (Fig. 5). These results indicate that other growth‐promoting peptide hormones can protect Arabidopsis from FB1 toxicity.

### PSKR1 and BAK1 are essential for flg22 to suppress FB1‐induced cell death

While our results demonstrate a clear role for PSK signalling in blocking FB1‐induced cell death, the natural conditions under which this mechanism is activated remain unclear. To explore this, we examined whether pattern‐triggered immunity (PTI), which is known to suppress FB1‐induced cell death, involves PSK signalling. Previous studies have shown that flg22, a bacterial flagellin‐derived peptide that activates PTI, prevents FB1‐induced cell death when applied 24 h before FB1 treatment (Igarashi *et al*., 2013). This prompted us to investigate whether flg22 might possibly block FB1 via activation of PSK signalling.

**Fig. 6:**
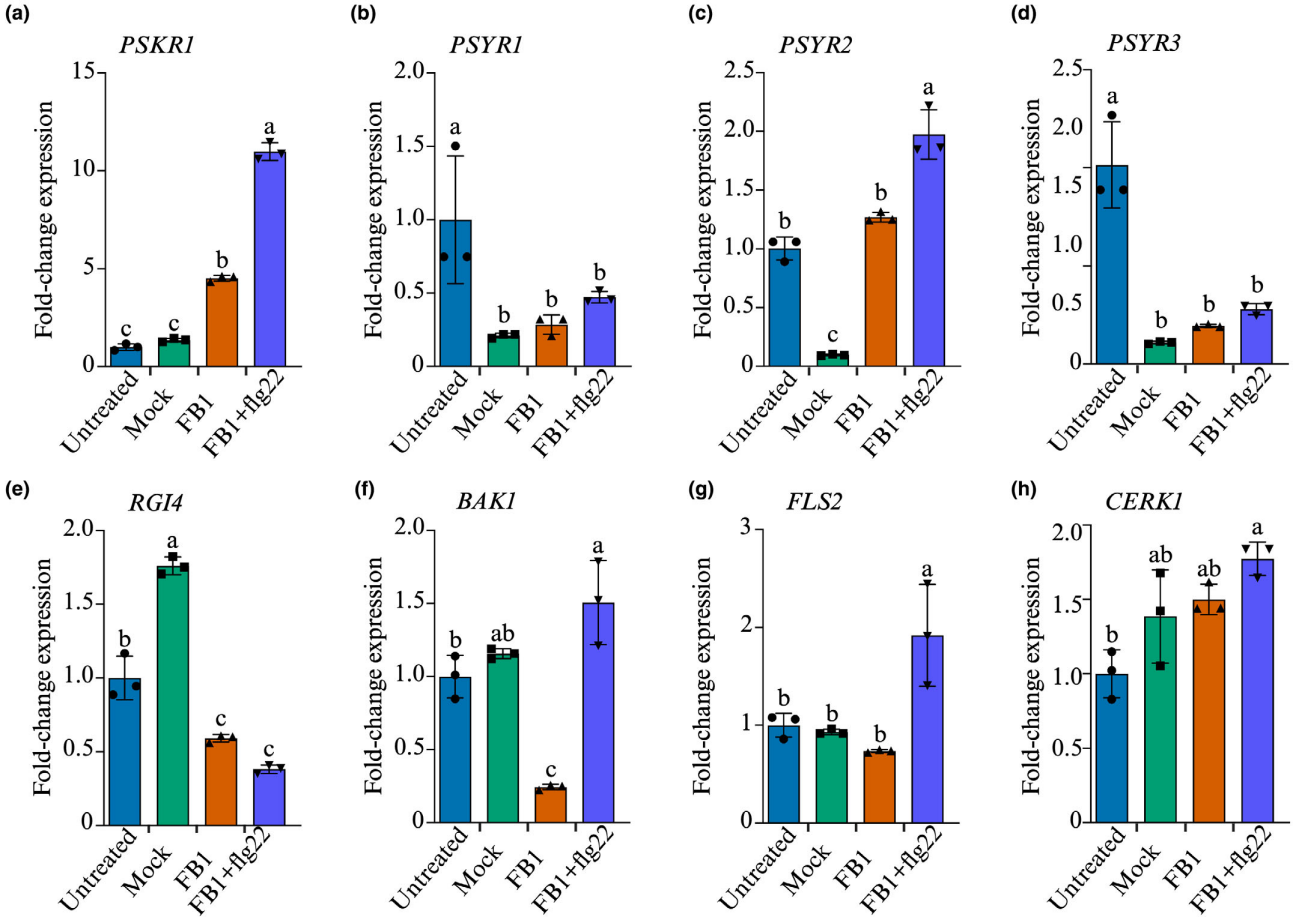
The impact of flg22 on the response of peptide receptor and coreceptor genes to FB1. Gene expression analysis of (a) *PSKR1*, (b) *PSYR1*, (c) *PSYR2*, (d) *PSYR3*, (e) *RGI4*, (f) *BAK1*, (g) *FLS2*, and (h) *CERK1* in untreated wild‐type Arabidopsis leaves, after mock treatment with a control carrier solution, or treatments with 5 μM FB1 ± 100 nM flg22. Samples were harvested at 48 h for qRT‐PCR analysis. Bars represent mean ± SD (*n* = 3). Data were analysed by one‐way ANOVA and Tukey test. Bars not sharing the same letters are significantly different (*P* ≤ 0.05). FB1 Fumonisin B1.

We first evaluated whether flg22 regulates the expression of PSK signalling genes. FB1 treatment alone caused a modest increase in *PSKR1* expression (Fig. 6a). However, co‐treatment with FB1 and flg22 led to a massive increase in *PSKR1* expression, which was more than 10‐fold higher than mock‐treated controls (Fig. 6a). Although FB1 modestly increases *PSKR1* expression, it significantly downregulates expression of the gene encoding the BAK1 co‐receptor (Fig. 6a,f), which inevitably suppresses PSK signalling to allow cell death activation. However, in addition to activating a surge in *PSKR1* expression, flg22 prevents *BAK1* suppression, ensuring that PSK signalling is enhanced (Fig. 6a,f). This raises the possibility that flg22 may block FB1‐induced cell death, at least partially, via stimulation of PSK signalling through the PSKR1–BAK1 receptor complex. However, without confirming that the gene expression results are reflected by corresponding protein expression, the speculative conclusions require validation by alternative approaches.

Since preceding results revealed that exogenous application of other sulfated peptide growth hormones can block FB1‐induced cell death, we wanted to check if flg22 equally activates genes encoding receptors for these alternative peptides. We analysed the expression of genes encoding PSY1 receptors: *PSYR1, PSYR2, PSYR3* (Ogawa‐Ohnishi *et al*., 2022), and the gene encoding the RGF7 receptor *RGI4* (Wang *et al*., 2021). In contrast to *PSKR1* and *BAK1*, the response of *PSYR1, PSYR3 and RGI4* to FB1 was unaltered by co‐treatment with flg22 (Fig. 6b,d,e). While co‐application of FB1 + flg22 modestly increased *PSYR2* to just below twofold, this was dwarfed by the over 10‐fold increase in *PSKR1*, suggesting that PSK‐PSKR1 signalling has the predominant role in the flg22‐triggered suppression of FB1 cell death. These results suggest that flg22 preferentially enhances PSK signalling over PSY1 or RGF7 signalling pathways.

Addition of flg22 with FB1 activated the gene encoding the flg22 receptor FLAGELLIN SENSITIVE 2 (FLS2; Fig. 6g) but not the gene encoding the fungal CHITIN ELICITOR RECEPTOR KINASE 1 (CERK1; Fig. 6h). Overall, these results support the notion that, in parallel to activation of PTI via FLS2–BAK1 signalling, flg22 concurrently suppresses FB1‐induced cell death through PSK signalling via the PSKR1–BAK1 complex. However, these results provide only correlative evidence suggesting that flg22‐mediated protection invokes PSK signalling. Definitive genetic evidence is required to warrant such a conclusion.

**Fig. 7:**
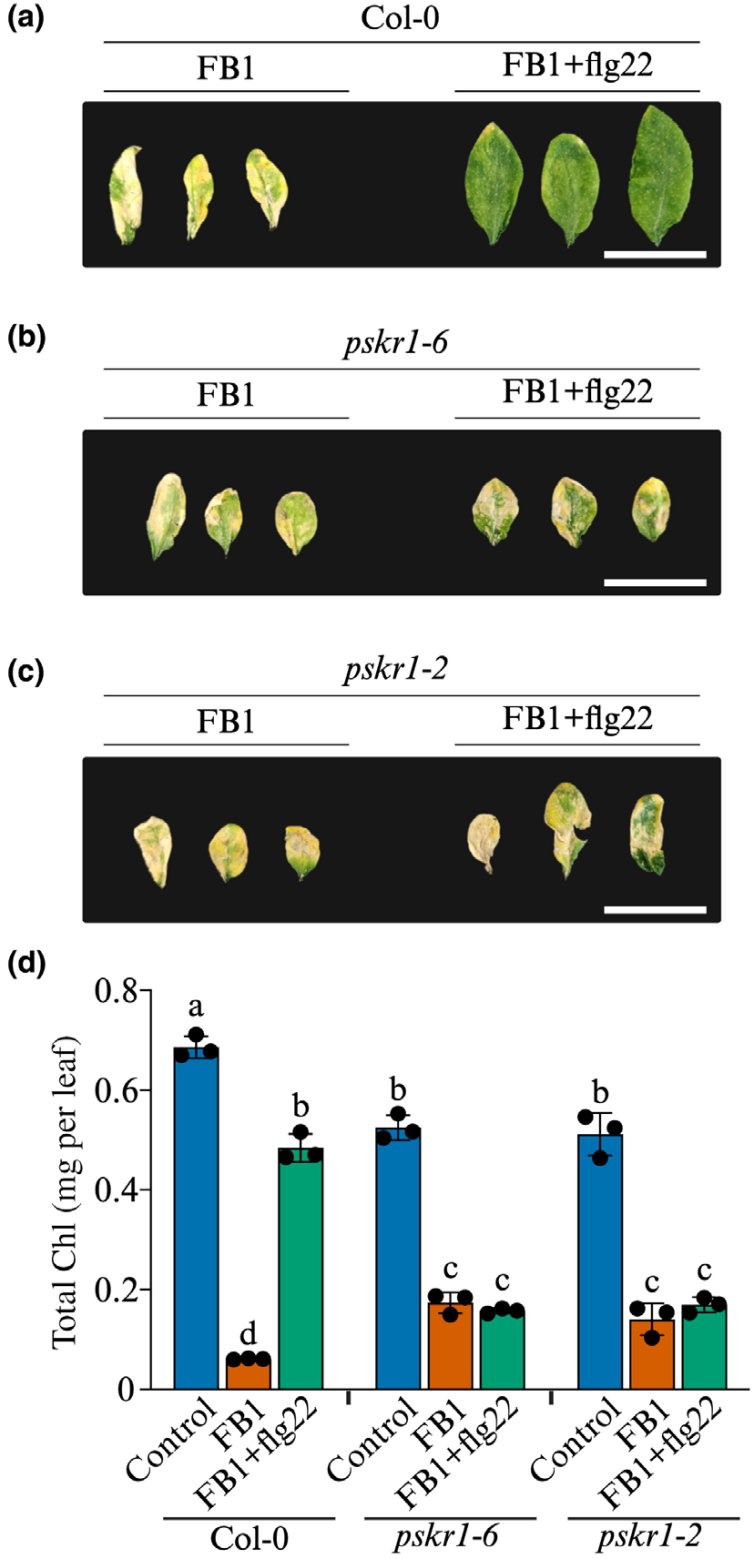
Flg22 requires PSKR1 to block FB1‐induced cell death. (a–c) Arabidopsis wild‐type and *pskr1* mutant plants were treated with 5 μM FB1 ± 100 nM flg22. Photographs of representative leaves were taken 7 d post‐treatment. Bars, 20 mm. (d) Chl content in leaves of Col‐0, *pskr1‐6*, and *pskr1‐2* plants was measured 7 d after exposure to FB1. Bars represent mean ± SD (*n* = 3). Statistical analysis was performed using 2‐way ANOVA and Tukey test. Bars not sharing the same letters are significantly different (*P* ≤ 0.05). Col‐0, Columbia‐0; FB1, Fumonisin B1; PSKR1, PSK RECEPTOR 1.

To investigate a potential role for PSK signalling downstream of flg22, we used *pskr1* knockout mutants. Wild‐type Col‐0 and *pskr1* mutants (*pskr1‐6* and *pskr1‐2*) were exposed to treatments with 5 μM FB1 ± 100 nM flg22. After 7 d, Col‐0 plants exposed to FB1 had severe tissue damage, but inclusion of flg22 blocked the cell death (Fig. 7a). However, flg22 failed to protect *pskr1* mutants from FB1 toxicity, as equally extensive cell death symptoms were observed in both FB1‐only treatments and FB1 + flg22 treatments (Fig. 7b,c). Unlike in Col‐0 plants, where flg22 attenuated cell death and Chl degradation (Fig. 7d), Chl degradation was not abated by flg22 in *pskr1* mutants (Fig. 7d). These results provide strong genetic evidence supporting the conclusion that PSK signalling is downstream of flg22‐dependent inhibition of FB1‐induced cell death.

To demonstrate the specificity of both flg22 and PSK for their receptors, we used *cerk1* mutants (Fig. S2) and Arabidopsis Wassilewskija‐0 (Ws‐0) ecotype or Ws‐0 transformed with the *FLAGELLIN SENSITIVE 2* (*FLS2*) gene from Col‐0. The Ws‐0 ecotype has a naturally occurring *fls2* loss‐of‐function mutation. Notably, flg22 failed to rescue wild‐type Ws‐0 plants from FB1 toxicity, confirming that flg22 requires its receptor FLS2 to block FB1 (Fig. S3). This was reinforced by the observation that flg22 fully rescued the transgenic Ws‐0 line expressing a functional FLS2 (Fig. S3). PSK fully rescued Ws‐0 plants from FB1 toxicity (Fig. S3). Flg22 rescued both wild‐type and *cerk1* mutants from FB1‐induced cell death (Fig. S4), showing that the flg22‐mediated rescue does not require CERK1 or any other PRR, except FLS2. PSK equally blocked FB1‐induced cell death in wild‐type and *cerk1* mutants (Fig. S4), indicating that PSK does not require CERK1. Overall, these results show that both flg22 and PSK work through their specific receptors to protect Arabidopsis from FB1.

### PSK prevents cell death induced by alternariol

**Fig. 8:**
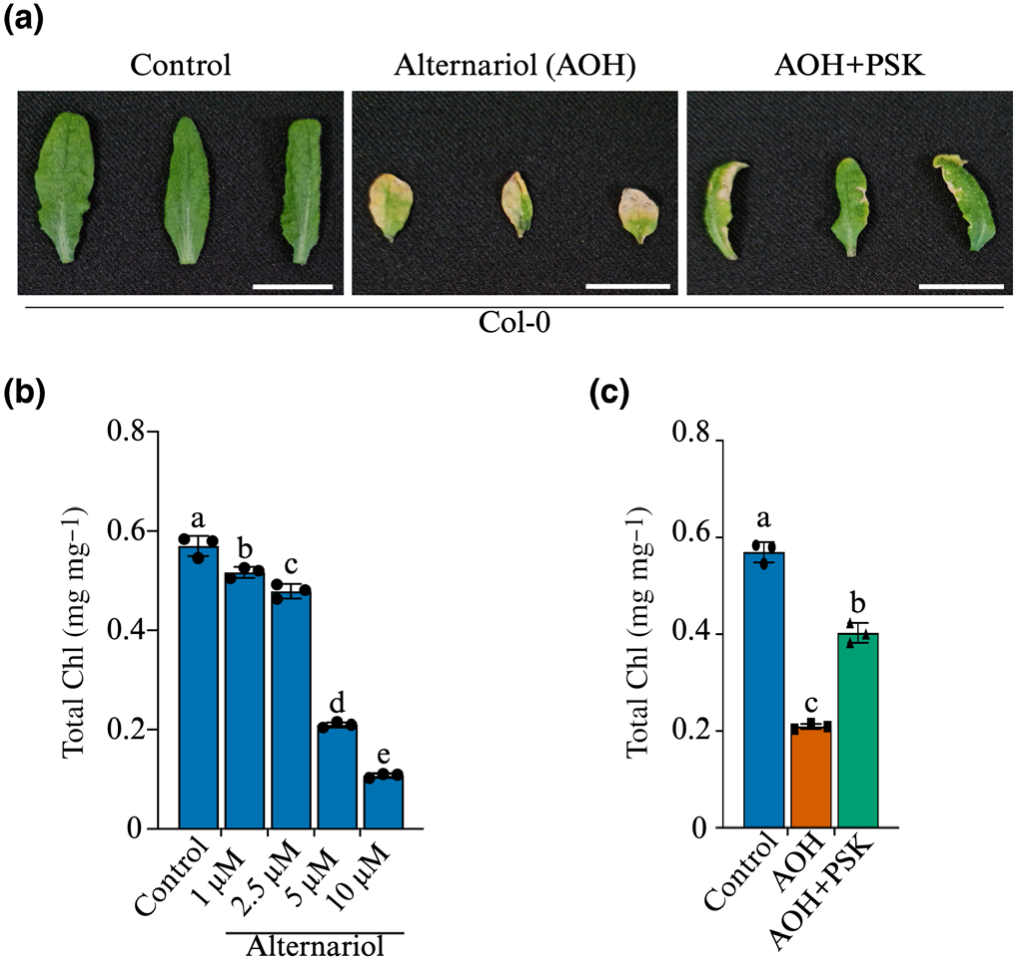
PSK attenuates alternariol (AOH)‐induced cell death in Arabidopsis. (a) Representative leaves of Col‐0 plants treated with 5 μM AOH ± 100 nM PSK or mock (control) treatment. Photographs were taken 7 d post‐treatment. Bars, 20 mm. (b) Chl content was measured in Col‐0 leaves treated with increasing concentrations of AOH for 7 d. (c) Chl content in leaves treated with 5 μM AOH ± 100 nM PSK for 7 d. Bars represent mean ± SD (*n* = 3). Statistical analysis was performed using one‐way ANOVA. Bars that do not share the same letter are significantly different (*P* ≤ 0.05). Col‐0, Columbia‐0; FB1 Fumonisin B1; PSK, phytosulfokine.

A previous study reported that ectopic expression of PSK terminates Arabidopsis infection by the necrotrophic fungus *Alternaria brassicicola* (Mosher *et al*., 2013). A key strategy of necrotrophic pathogens is the secretion of mycotoxins. Alternariol (AOH) is a major mycotoxin of *A. brassicicola* and other *Alternaria* species. Therefore, we used AOH to investigate if PSK signalling blocks cell damage by a mycotoxin from an Arabidopsis pathogen. We first established the dose–response profile of AOH in Arabidopsis. There was a dose‐dependent decline in Chl content, with higher toxin levels triggering drastic Chl destruction (Fig. 8a,b). Based on this, we selected 5 μM AOH for subsequent experiments. Leaves of Col‐0 plants were treated with 5 μM AOH ± 100 nM PSK. AOH caused significant cell death, characterised by leaf chlorosis and necrosis 7 d post treatment (Fig. 8a). Co‐treatment with PSK reduced these symptoms, effectively blocking widespread death (Fig. 8a), as confirmed by Chl quantification (Fig. 8c). These results suggest that PSK signalling has a broad‐spectrum protective role, which probably operates against a wide range of mycotoxins. Importantly, this suggests that exogenous PSK termination of Arabidopsis infection by *A. brassicicola* (Mosher *et al*., 2013) integrated suppression of phytotoxicity, since mycotoxins are critical virulence factors for necrotrophs.

### Impact of FB1 and PSK on the Arabidopsis proteome

We used proteomic analysis to gain insight into how exogenous PSK rescues Arabidopsis from FB1‐induced cell death. We decided to use tissue culture‐grown plants to capture proteomic changes in both roots and shoots. While we consistently used 5 μM FB1 to treat soil‐grown plants, we had to conduct a dose–response experiment to identify the appropriate amount of FB1 for use in agar plates. The selected concentration was 100 nM FB1, which enabled germination of surface‐sterilised seeds and allowed sufficient seedling growth to obtain enough biomass (Fig. S5). Therefore, surface‐sterilised seeds were germinated and grown for 15 d on agar plates with carrier solution (controls), 100 nM PSK or 100 nM FB1 ± 100 nM PSK. This timepoint was selected because the plants across all treatments had a well‐developed rosette and sufficient biomass for analysis (Fig. S6). Although the FB1 concentration used was not sufficient to curb root length, it significantly reduced root thickness, and the inclusion of PSK in the treatment abolished this reduction in root thickness (Fig. S6). Furthermore, PSK treatments with or without FB1 stimulated root lengthening (Fig. S6), demonstrating that the tissue culture plants responded to the treatments as expected. We selected three treatments for further proteomics analysis: control, FB1, and FB1 + PSK. Protein was extracted from four replicates of each treatment, and trypsin digests labelled for iTRAQ analysis. A nonredundant list of 888 proteins was generated by filtering the data to retain candidates identified based on ≥ 2 peptides sequenced at ≥ 95% confidence level (Table S1). After statistical analysis, a total of 67 proteins were significantly changed (*P* ≤ 0.05) in response to FB1, while the abundance of 68 proteins differed between FB1 and FB1 + PSK treatments (Table S2). These two protein categories gave a combined total of 117 nonredundant responsive proteins.

Three clear trends emerged from the protein datasets (Tables 1, S2). First, there were 49 proteins that responded to FB1 treatment, with the inclusion of PSK having no significant impact on their FB1 response. These proteins are clearly not PSK targets in blocking FB1‐induced cell death. Second, there were 18 proteins whose response to FB1 was significantly changed by PSK. Third, there were 50 proteins that did not respond significantly to FB1 but were activated/suppressed by the combined FB1 + PSK treatment (Tables 1, S2). The expression profile of the latter two categories is consistent with proteins expected to be putative PSK targets. However, it is striking that the impact of PSK on the plant proteome was predominantly on proteins not directly affected by FB1 (50 proteins), and relatively limited (18 proteins) on FB1‐responsive proteins. Notably, the inclusion of PSK in treatments significantly attenuated or completely blocked the FB1 response of 14 of these 18 proteins (Table S3). These results may suggest that during cell death suppression, PSK integrates stimulation of PSK‐specific targets with direct antagonistic action at downstream protein targets common to both FB1 and PSK signalling. A heatmap was used to provide a visual summary of the proteomic changes across the treatments (Fig. S7).

**Table 1:** List of the top 50 Arabidopsis proteins differentially expressed in response to FB1 ± PSK treatments.

Locus^a^	Protein name	FB1/Control		FB1/FB1 + PSK	
		Ratio^b^	*P*‐value^c^	Ratio^d^	*P*‐value^c^
AT3G12780	Phosphoglycerate kinase 1, chloroplast	−1.00	0.78	1.35	0.01
AT5G53560	Cytochrome b5 isoform E	−1.11	0.25	1.32	0.01
AT5G35100	Peptidyl‐prolyl *cis*‐*trans* isomerase, chloroplast	−1.34	0.01	1.52	0.79
AT5G66530	Aldose 1‐epimerase family protein	−1.17	0.00	1.51	0.23
AT5G38410	Ribulose bisphosphate carboxylase small chain 3B, chloroplast	−1.00	0.93	1.43	0.02
AT3G52300	ATP synthase subunit d, mitochondrial	1.03	0.23	−1.43	0.00
AT4G09010	Thylakoid lumenal 29 kDa protein, chloroplast	−1.01	0.24	1.42	0.01
AT1G67090	Ribulose bisphosphate carboxylase small chain 1A, chloroplast	−1.08	0.40	1.41	0.01
AT1G16080	Putative uncharacterised protein	−1.30	0.06	−1.31	0.00
AT5G58250	YCF54	−1.29	0.73	1.30	0.01
AT4G12060	ATP‐dependent Clp protease ATP‐binding subunit, chloroplast	−1.36	0.19	1.30	0.04
AT1G06680	Oxygen‐evolving enhancer protein 2‐1, chloroplast	−1.22	0.01	1.29	0.01
AT3G18890	Protein TIC 62, chloroplast	−1.02	0.25	1.26	0.00
AT2G20690	Lumazine‐binding family protein	−1.12	0.10	1.26	0.01
AT3G54050	Fructose‐1,6‐bisphosphatase 1, chloroplast	−1.21	0.01	1.25	0.03
AT1G13440	Glyceraldehyde‐3‐phosphate dehydrogenase, cytosolic	−1.04	0.10	−1.24	0.05
AT4G04640	ATP synthase gamma chain 1, chloroplast	−1.02	0.83	1.23	0.05
AT1G12840	V‐type proton ATPase subunit C	−1.06	0.83	−1.23	0.01
ATCG00490	Ribulose bisphosphate carboxylase large chain	−1.12	0.20	1.22	0.03
AT2G46820	Protein CURVATURE THYLAKOID 1B, chloroplast	−1.52	0.03	1.21	0.02
AT3G50820	Oxygen‐evolving enhancer protein 1‐2, chloroplast	−1.20	0.05	1.19	0.01
AT2G28190	Superoxide dismutase 2, chloroplast	1.40	0.00	1.66	0.10
AT2G14610	Pathogenesis‐related protein 1	1.91	0.01	−1.70	0.08
AT5G14200	3‐Isopropylmalate dehydrogenase 3, chloroplast	1.01	0.89	1.40	0.05
AT2G28000	Chaperonin 60 subunit alpha 1, chloroplast	1.21	0.02	1.39	0.05
AT5G47210	RGG repeats nuclear RNA‐binding protein C	1.30	0.01	−1.39	0.03
AT1G56070	Elongation factor 2	1.05	0.57	−1.36	0.01
AT1G78300	14–3‐3‐like protein GF14 omega	1.17	0.16	−1.31	0.04
AT1G65960	Glutamate decarboxylase 2	1.41	0.05	1.31	0.01
ATCG00120	ATP synthase subunit alpha, chloroplast	1.13	0.26	1.30	0.02
AT1G55490	Chaperonin 60 subunit beta 1, chloroplast	1.12	0.07	1.30	0.01
AT4G28750	Photosystem I reaction centre subunit IV A, chloroplast	1.18	0.03	−1.29	0.48
AT4G09320	Nucleoside diphosphate kinase 1	1.05	0.92	1.29	0.01
AT5G35630	Glutamine synthetase, chloroplast/mitochondrial	1.44	0.00	1.29	0.01
AT5G50920	Chaperone protein ClpC1, chloroplast	1.27	0.19	−1.29	0.04
AT5G02790	Glutathione S‐transferase	1.11	0.01	1.27	0.74
AT2G27020	Proteasome subunit alpha type 3	1.07	0.54	1.27	0.03
AT4G11150	V‐type proton ATPase subunit E1	1.03	0.18	−1.26	0.00
AT5G39570	Uncharacterised protein	1.02	0.86	−1.26	0.01
AT2G44060	Late embryogenesis abundant 26	1.03	0.31	1.25	0.00
ATMG01190	ATP synthase subunit alpha, mitochondrial	1.52	0.01	−1.24	0.16
AT3G14930	Uroporphyrinogen decarboxylase 1, chloroplast	1.16	0.02	1.24	0.12
AT4G27520	Early nodulin‐like protein 2	1.01	0.81	1.24	0.02
AT3G57610	Adenylosuccinate synthetase, chloroplast	1.13	0.21	−1.23	0.01
AT2G21170	Triosephosphate isomerase, chloroplast	1.18	0.00	1.23	0.02
AT3G59970	Methylenetetrahydrofolate reductase 1	1.06	0.49	−1.22	0.02
AT5G20720	20 kDa chaperonin, chloroplast	1.09	0.02	1.22	0.04
AT1G24020	MLP‐like protein 423	1.12	0.31	−1.21	0.03
AT5G48300	Glucose‐1‐phosphate adenylyltransferase small subunit, chloroplast	1.31	0.00	1.21	0.03
AT3G61440	L‐3‐cyanoalanine synthase/cysteine synthase C1, mitochondrial	1.32	0.03	−1.19	0.15

The top 50 proteins were selected by first filtering out proteins with insignificant statistical scores (*P* > 0.05). The proteins with significant scores were ranked in descending order of the magnitude of change and the top 50 between the FB1 and FB1 + PSK lists were selected for inclusion in the table. FB1, fumonisin B1; PSK, phytosulfokine.

^a^ Locus identifier as given in the TAIR database (https://www.arabidopsis.org/).

^b^ Ratio is the average fold change (*n* = 3) induced by FB1 relative to the control. Negative values indicate a decrease in protein abundance.

^c^ Probability value of the quantitative difference between two groups under comparison arising due to chance alone, and not treatment effects.

^d^ Ratio is the average fold change (*n* = 3) induced by FB1 + PSK relative to the FB1 treatment. Negative values indicate a decrease in protein abundance.

The magnitude of protein abundance change across the dataset was relatively modest, with the top protein at 1.91‐fold change (Table 1). This was not unexpected, given that we used a sublethal concentration of FB1 to conduct the experiment. The aim was to identify metabolic pathways responsive to the mycotoxin concentration (100 nM FB1), which is 50‐fold lower than the 5 μM rate used to activate Arabidopsis death. Our hypothesis was that the proteome changes allowing plants to adapt to the low FB1 concentration would be important targets for FB1 and PSK in controlling cell viability.

### The Calvin cycle is a major target of FB1 and PSK signalling

**Fig. 9:**
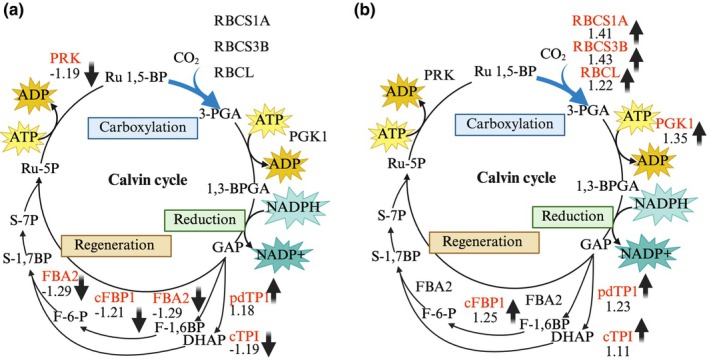
Schematic presentation of the Calvin cycle. (a) Proteins that respond significantly (*P* ≤ 0.05) to FB1 are in red font. (b) Proteins significantly different (*P* ≤ 0.05) between FB1 and FB1 + PSK are indicated in red font. Values and arrows show the direction and magnitude of the fold‐change response. 1,3‐BPGA, 1,3‐Bisphosphoglycerate; 3‐PGA, 3‐Phosphoglyceric acid; cFBP1, chloroplast FBPase 1; cTPI, cytosolic triose phosphate isomerase; DHAP, dihydroxyacetone phosphate; F‐1,6BP, fructose‐1,6‐bisphosphate aldolase; F‐6‐P, fructose 6‐phosphate; FBA2, fructose‐bisphosphate aldolase 2; GAP, glyceraldehyde 3‐phosphate; pdTPI, plastid triose phosphate isomerase; PGK1, phosphoglycerate kinase 1; PRK, phosphoribulokinase; RBCL, ribulose bisphosphate carboxylase large subunit; RBCS1A/RBCS3B, ribulose bisphosphate carboxylase small chain 1A/3B; Ru 1,5‐BP, ribulose 1,5‐bisphosphate (RuBP); Ru‐5P, ribulose 5‐phosphate; S‐1,7BP, sedoheptulose 1,7‐bisphosphate; S‐7P, sedoheptulose 7‐phosphate. This figure was created in BioRender (Alqarni, A. (2025) https://BioRender.com/538axah).

In response to FB1 treatment, proteins localised across many cellular compartments, including the cytosol, chloroplasts, mitochondria, the nucleus and apoplast, were differentially expressed (Table S2), reflecting the widespread impact of FB1. Gene Ontology (GO) enrichment analysis performed using shinygo v.0.82 (Ge *et al*., 2020) indicated a high representation of Calvin cycle‐related processes, specifically focusing on the reduction of the pentose‐phosphate cycle, dark reaction, and carbon fixation (Fig. S8). Given its predominance in the proteome data, we accordingly focused on the Calvin cycle and associated regulatory proteins to understand how PSK signalling averts FB1‐induced cell death. Therefore, we generated schematic diagrams of the Calvin cycle and annotated proteins responding to treatments with FB1 or FB1 + PSK (Fig. 9). It became clear that FB1 had an overall effect of decreasing specific Calvin cycle enzymes, while the addition of PSK increased the enzymes (Fig. 9). Thus, phosphoribulokinase (PRK), fructose‐bisphosphate aldolase 2 (FBA2), fructose‐bisphosphatase 1 (FBP1), and cytosolic triose phosphate isomerase (cTPI) were decreased by FB1 (Fig. 9). Inclusion of PSK within the treatment increased cTPI, FBP1, Rubisco large subunit (RBCL), Rubisco small subunits 1A (RBCS1A) and 3B (RBCS3B), and phosphoglycerate kinase 1 (PGK1) (Fig. 9).

Proteins that regulate the Calvin cycle were also targeted by FB1 ± PSK treatments. For example, FB1 suppressed thioredoxin M1 (At1g03680) (Table S1), which is a member of the thioredoxin M family that reductively activates Calvin cycle enzymes (Okegawa & Motohashi, 2015). By contrast, PSK stimulated the expression of this protein (Table S1). Furthermore, PSK increased CP12 (Table S1), a key protein that interacts with phosphoribulokinase (PRK) and GAPDH to regulate the Calvin cycle (Elena López‐Calcagno *et al*., 2017). The respective changes in Calvin cycle enzymes and Calvin cycle regulatory proteins suggest that FB1 restricts the generation of metabolic building blocks, while PSK averts inhibition. This may account for the reported growth inhibition caused by FB1 treatments (Chivasa *et al*., 2005; Smith *et al*., 2015) and growth stimulation promoted by PSK (Matsubayashi & Sakagami, 1996; Stührwohldt *et al*., 2011), as also reflected in our results (Fig. S1).

### Rubisco is regulated by FB1 and PSK

**Fig. 10:**
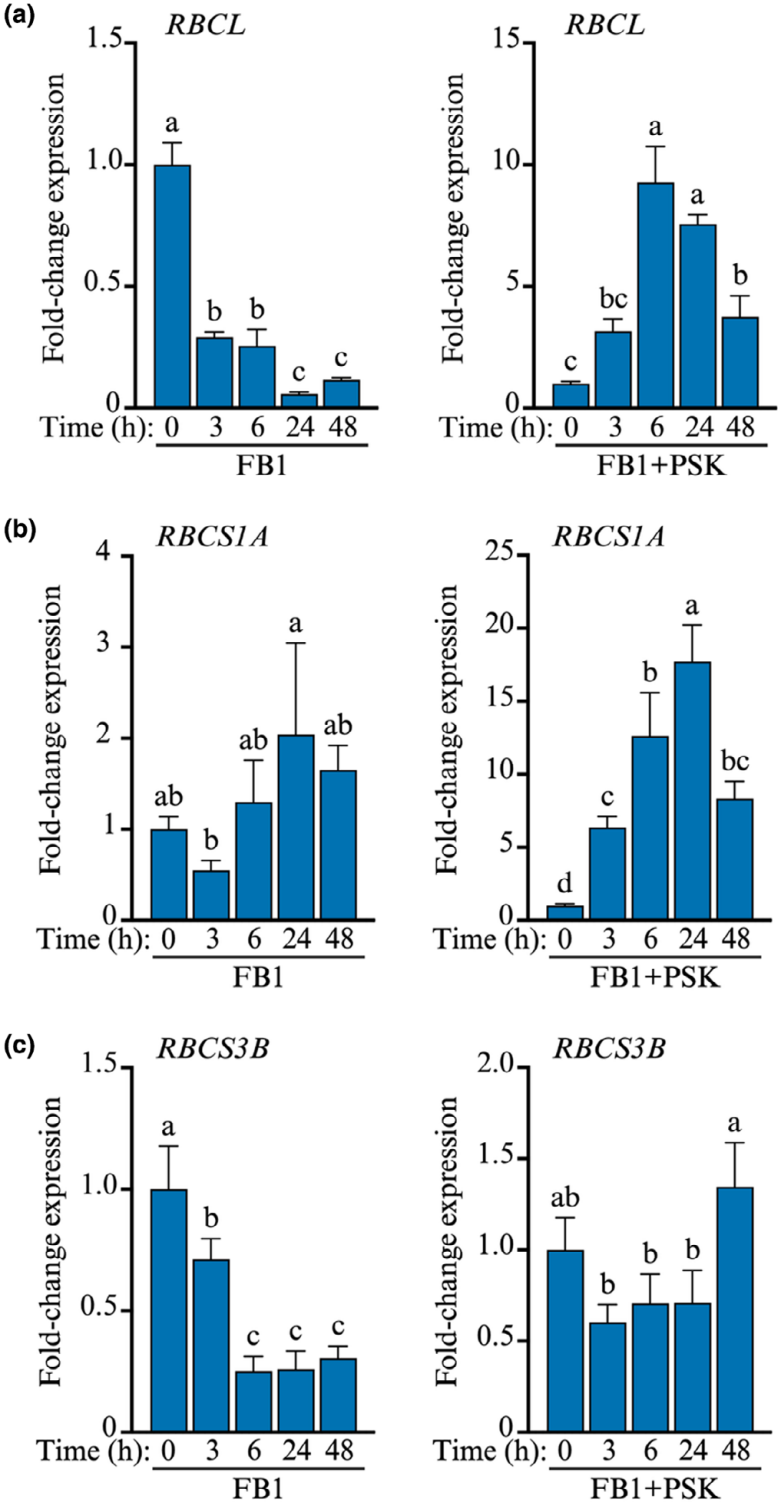
Effects of PSK on FB1‐induced gene expression. Leaves of Arabidopsis plants were infiltrated with 5 μM FB1 ± 100 nM PSK. Samples were harvested at the indicated timepoints for RNA extraction and qRT‐PCR analysis. Bar graphs show expression profiles of the genes encoding (a) RUBISCO ‐ LARGE SUBUNIT (RBCL), (b) RUBISCO ‐ SMALL SUBUNIT 1A (RBCS1A), and (c) RUBISCO ‐ SMALL SUBUNIT 3B (RBCS3B). Bars represent mean ± SD (*n* = 3) and bars not sharing the same letter are significantly different (*P* ≤ 0.05). Statistical analysis was performed using one‐way ANOVA. PSK, phytosulfokine; FB1 Fumonisin B1.

Since the Calvin cycle was identified as a target of FB1 in preceding experiments that used a sublethal dose, we examined the response of the pathway to a lethal dose of FB1 and focused on the rate‐limiting step catalysed by Rubisco. Although a lethal dose of FB1 was used, samples for RNA extraction were harvested in the 0–48‐h time window, before symptom development and onset of cell death. Three subunit proteins of the Rubisco holoenzyme: RBCL, RBCS1A, and RBCS3B, were selected for qRT‐PCR analysis. Leaves of Arabidopsis plants were infiltrated with 5 μM FB1 or 5 μM FB1 + 100 nM PSK, and samples harvested at different timepoints. There was a drastic shutdown of *RBCL* gene expression in response to FB1, but inclusion of PSK massively upregulated gene expression (Fig. 10a,b). Although RBCS1A expression was not significantly affected by FB1, it was also massively upregulated in the FB1 + PSK co‐treatment (Fig. 10c,d). FB1 suppressed *RBCS3B*, but expression was restored in the co‐treatment (Fig. 10e,f). These results align with proteomic data (Table 1; Fig. 9). Given that RBCL is encoded by a single copy gene in Arabidopsis, these results show that the Calvin cycle is indeed likely constricted by FB1, while PSK upregulates genes encoding subunits of the holoenzyme catalysing the rate‐limiting step of the Calvin cycle. Although these differences are seen way before the onset of cell death, further experiments would be needed to confirm if the changes to RBCL are a reflection of changes in the catalytic activity of the entire biochemical pathway.

## Discussion

Our study establishes PSK signalling as a key regulator of the Arabidopsis response to mycotoxin exposure. Pharmacological experiments demonstrated that exogenous PSK blocks mycotoxin‐induced cell death, definitively identifying the peptide as a negative regulator of the phytotoxic effects of mycotoxins. The suppression of stress‐induced cell death by an extracellular peptide highlights the vital role of the apoplast in integrating cellular life‐and‐death signals. This function is not without precedent in plants. For example, the extracellular PLANT NATRIURETIC PEPTIDE A (PNP‐A) binds its plasma membrane‐localised receptor, PNP–AR2, to abrogate salicylic acid‐primed cell death in Arabidopsis (Lee *et al*., 2020). Exogenous application of PNP‐A similarly blocks the runaway cell death phenotype observed in *lsd* loss‐of‐function mutants (Lee *et al*., 2020). Control of cell viability through extracellular signalling, as seen with both PSK and PNP‐A, points to a global role for the apoplast as a key compartment integrating diverse signals to activate or suppress cell death (Chivasa & Goodman, 2020). This idea is further supported by the existence of numerous proteins (Smith *et al*., 2015, 2021; Goodman *et al*., 2022) and peptides (Wrzaczek *et al*., 2015; Escamez *et al*., 2019) within the plant extracellular matrix that regulate stress‐induced or developmental cell death. For instance, the extracellular GRIM REAPER protein is cleaved to release a peptide signal that binds to POLLEN‐SPECIFIC RECEPTOR‐LIKE KINASE 5 (PRK5) to activate cell death (Wrzaczek *et al*., 2015). Taken together, our finding regarding PSK's protective role, combined with these previous reports, strongly supports the proposed functional role of the apoplast in executing life‐and‐death ‘decisions’ within plant tissues.

Genetic evidence strongly supports the role of PSK signalling in regulating Arabidopsis stress responses to FB1. Loss‐of‐function *sbt3.8* mutants, which are incapable of processing the full‐length PSK polypeptide into the functional pentapeptide, displayed increased sensitivity to FB1 toxicity. Furthermore, exogenous PSK failed to rescue FB1‐treated *pskr1* and *bak1* mutants, establishing a functional requirement for the PSKR1–BAK1 co‐receptor complex in mediating this protective response. This finding is consistent with the established role of BAK1 in regulating cell death, often functioning redundantly with other receptor‐like kinases. For instance, the Arabidopsis *bak1*/*bkk1* (*serk4*) (BAK1‐Like1/SERK4) double knockout mutant exhibits spontaneous cell death accompanied by constitutive defence gene expression and the accumulation of reactive oxygen species (He *et al*., 2007; de Oliveira *et al*., 2016). Similarly, the absence of the BAK1–BIK1 (BOTRYTIS‐INDUCED KINASE 1) complex in *bak1*/*bik1* double knockout mutants triggers constitutive immune responses and spontaneous cell death associated with severe growth defects (Liu *et al*., 2017). Our study establishes the importance of the BAK1–PSKR1 co‐receptor duo in mediating cell death reduction during mycotoxin stress. By linking a specific peptide‐receptor pathway (PSK–PSKR1/BAK1) to FB1 response, our data suggest that peptide signalling may underpin these previously published, broader cell death regulatory functions of BAK1.

Evaluation of the Arabidopsis proteome response following FB1 treatment revealed a specific targeting of the Calvin cycle, marked by the decrease of key catalytic enzymes and regulatory proteins (Table 1). This effect may be mediated by the chloroplast protease systems, specifically the caseinolytic protease (Clp) in the stroma and the filamentation‐temperature‐sensitive protein H (FtsH) protease system associated with thylakoid membranes (Wagner *et al*., 2012; S. Zhang *et al*., 2018). FB1 treatment led to an increased abundance of the FtsH2 protease and ClpP4 (Table 1). Notably, overexpression of ClpP4 in Arabidopsis plants is known to spontaneously induce chlorosis (Shen *et al*., 2007) – a symptom strikingly similar to that caused by FB1. Conversely, the application of PSK blocked the increase in both these proteases and also caused a decrease in ClpC1 (Table 1), a subunit critical for the chaperon ring of the Clp protease. While FB1 is known to inhibit ceramide synthase (Wang *et al*., 1991; Merrill Jr *et al*., 1993) and disrupt sphingolipid biosynthesis, triggering cell death in both animal and plant cells (Gilchrist *et al*., 1992; Huang *et al*., 1995), the precise mechanism of cell death activation in plants remains unclear. Exogenous ceramide rescues animal cells from FB1‐induced death (Harel & Futerman, 1993) but fails to rescue plant cells (Stone *et al*., 2000). Our results, which identify photosynthesis as a key target process in plants, offer a potential explanation for the observations by Stone *et al*. (2000) and also account for the requirement of light for FB1 to fully activate cell death in plants.

While previous research established that flg22 treatment suppresses FB1‐induced cell death (Igarashi *et al*., 2013), our study presents novel insights by detailing the involvement of PSK signalling. We reveal the mechanism by which flg22, a bacterial microbe‐associated molecular pattern (MAMP), blocks FB1‐induced cell death: flg22 activates PSKR1 gene expression and blocks the downregulation of BAK1, thereby increasing the capacity for PSK signalling. Crucially, flg22 did not activate genes encoding the related receptors PSYR1, PSYR2, PSYR3, and RGI4, underscoring the specificity of PSK signalling in this protective context.

Our finding that bacterial MAMP perception activates *PSKR1* expression to suppress mycotoxin‐induced cell death provides a mechanistic understanding of previously published data on defence priming. For instance, flg22 pretreatment in Arabidopsis abrogates cell death caused by the necrotrophic fungus *Botrytis cinerea* (Ferrari *et al*., 2007; Galletti *et al*., 2011), a pathogen known to secrete cell death‐activating mycotoxins (Huo *et al*., 2018). The involvement of PSK is evident: exogenous PSK reduces *B. cinerea* infection of tomato plants, while loss‐of‐function *pskr1* mutants exhibit enhanced infection (H. Zhang *et al*., 2018; Ding *et al*., 2024). Similarly, silencing genes for PSK precursor peptides or TYROSYLPROTEIN SULFOTRANSFERASE (TPST) increases the severity of *B. cinerea* infection (H. Zhang *et al*., 2018). Given the established contribution of mycotoxins to necrotrophic pathogen virulence, these reports, combined with our findings, strongly suggest that flg22 suppression of fungal infection includes the underlying mechanism of PSK cell death suppression. For this to represent a conserved defence mechanism, PSK signalling must antagonise cell death activated by multiple mycotoxins – beyond FB1.

Our data, showing that exogenous PSK abrogates alternariol (AOH)‐induced cell death, successfully expands the range of necrotrophic fungi against which activated PSK signalling is effective. AOH is a major cell death‐activating mycotoxin produced by *Alternaria* species (Wenderoth *et al*., 2019). The necessity of PSK signalling in basal resistance is further underscored by the following genetic evidence: Arabidopsis loss‐of‐function *pskr1* and *tpst* mutants displayed runaway cell death and spreading infection when inoculated with *Alternaria brassicicola* (Mosher *et al*., 2013). Conversely, ectopic expression of PSK precursor peptides suppressed infection by *A. brassicicola* (Mosher *et al*., 2013). Overall, these reports and our study reveal that bacterial MAMP activation of PSK signalling governs plant cell death and subsequent interactions with necrotrophic fungi.

The direct connection between flg22 and PSK signalling, revealed by our study, has significant ecological relevance by explaining observed protection phenomena. Plants are continuously threatened by necrotrophic pathogens that use mycotoxins to kill host cells. The established role of the bacterial MAMP–PSK signalling axis in blocking mycotoxin‐induced cell death provides a robust mechanistic explanation for the protection conferred by plant growth‐promoting bacteria (PGPB) against necrotrophic fungi. For example, prophylactic application of *Bacillus cereus* AR156 suppresses *B. cinerea*‐activated necrosis in Arabidopsis (Nie *et al*., 2017), and pretreatment with the PGPB *Pseudomonas fluorescens* WCS417r protects Arabidopsis from *Alternaria brassicicola* damage (Ton *et al*., 2002). We speculate that PGPB‐borne MAMPs trigger the PSK‐mediated protective mechanisms, resulting in enhanced resistance.

In summary, our study reveals the molecular process underpinning the antagonistic relationship between FB1 and the PSK peptide. While FB1 inhibits plant growth and cell proliferation (Chivasa *et al*., 2005), PSK promotes them; concurrent application shows that PSK effectively abolishes all FB1‐driven detrimental effects. This antagonism converges on the regulation of the Calvin cycle. Our data demonstrate that plants have adapted to utilise PSK signalling as a molecular lever to evade the debilitating impact of mycotoxins. More broadly, integrating bacterial MAMP perception as a signal to activate PSK protection provides the ecological rationale for harnessing the soil microbiome (particularly PGPB) as a broad‐spectrum defence strategy against necrotrophic fungi. Given the economic importance of mycotoxin‐producing pathogens, these findings could be exploited to enhance disease resistance by manipulating Calvin cycle regulation. Future research must focus on identifying the direct PSKR1 targets responsible for stimulating the Calvin cycle to fully harness this adaptive defence strategy.

## Competing interests

None declared.

## Author contributions

SC designed and supervised the research, AOA and JMUH conducted experiments, APB performed LC–MS/MS analysis, and the manuscript was written by AOA and SC.

## Disclaimer

The New Phytologist Foundation remains neutral with regard to jurisdictional claims in maps and in any institutional affiliations.

## Supporting information


**Fig. S1** PSK promotes growth of FB1‐treated leaves.
**Fig. S2** Genotypes of *pskr1*, *bak1*, *cerk1* and *sbt3.8* mutants.
**Fig. S3** Effects of FLS2 on the cell death‐inhibitory functions of PSK and flg22.
**Fig. S4** CERK1 is not required for the PSK and flg22 function in blocking FB1 toxicity.
**Fig. S5** Dose–response effects of FB1 on Arabidopsis growth.
**Fig. S6** Effects of FB1 and PSK on Arabidopsis growth.
**Fig. S7** Heatmap view of Arabidopsis proteome response to FB1 and PSK treatments.
**Fig. S8** Biological process Gene Ontology enrichment analysis.

**Table S1** Peptide identification data for control and FB1‐treated and peptide identification data for samples treated with FB1 ± PSK.
**Table S2** Full list and quantification data of differentially expressed proteins.
**Table S3** List of the 18 expressed proteins in both FB1 and FB1 + PSK treatments.
Please note: Wiley is not responsible for the content or functionality of any Supporting Information supplied by the authors. Any queries (other than missing material) should be directed to the *New Phytologist* Central Office.

## Data Availability

Additional data can be found in the Supporting Information (Tables S1–S3).
