# Supplementary material for: Phytosulfokine signalling blocks mycotoxin toxicity in Arabidopsis and mediates suppression of cell death activated by bacterial microbe‐associated molecular patterns
Source: New Phytol. 2025 Dec 12;249(5):2515–30. doi: 10.1111/nph.70811 (PMC12873527; doi:10.1111/nph.70811)
Supplement: Supplementary file 1 — Fig. S1 PSK promotes growth of FB1‐treated leaves. Fig. S2 Genotypes of pskr1, bak1, cerk1 and sbt3.8 mutants. Fig. S3 Effects of FLS2 on the cell death‐inhibitory functions of PSK and flg22. Fig. S4 CERK1 is not required for the PSK and flg22 function in blocking FB1 toxicity. Fig. S5 Dose–response effects of FB1 on Arabidopsis growth. Fig. S6 Effects of FB1 and PSK on Arabidopsis growth. Fig. S7 Heatmap view of Arabidopsis proteome response to FB1 and PSK treatments. Fig. S8 Biological process Gene Ontology enrichment analysis. [file NPH-249-2515-s001.pdf]

New Phytologist **Supporting Information**

Article title: Phytosulfokine signalling blocks mycotoxin toxicity in Arabidopsis and mediates suppression of cell death activated by bacterial microbe-associated molecular patterns
Authors: Ali O. Alqarni, John M. U. Hamilton, Adrian P. Brown, and Stephen Chivasa
Article acceptance date: 17 November 2025

The following Supporting Information is available for this article:

Figure S1. PSK promotes growth of FB1-treated leaves.

Figure S2. Genotypes of *pskr1*, *bak1*, *cerk1*, and *sbt3.8* mutants.

Figure S3. Effects of FLS2 on the cell death-inhibitory functions of PSK and flg22.

Figure S4. CERK1 is not required for the PSK and flg22 function in blocking FB1 toxicity.

Figure S5. Dose-response effects of FB1 on Arabidopsis growth.

Figure S6. Effects of FB1 and PSK on Arabidopsis growth.

Figure S7. Heatmap view of Arabidopsis proteome response to FB1 and PSK treatments.

Figure S8. Biological process Gene Ontology enrichment analysis.

Table. S1a Peptide identification data for control and FB1-treated.

Table. S1b Peptide identification data for samples treated with FB1±PSK.

Table. S2 Full list and quantification data of differentially expressed proteins.

Table. S3 List of the 18 expressed proteins in both FB1 and FB1+PSK treatments.

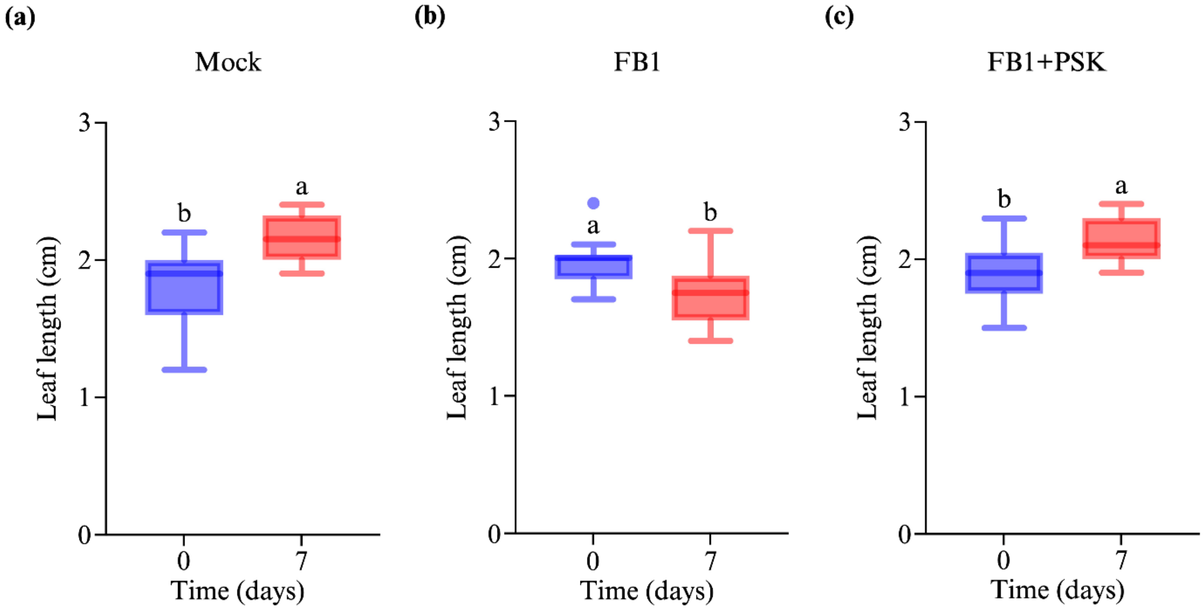

Figure S1. PSK promotes growth of FB1-treated leaves. The lengths of Col-0 leaves were measured prior to infiltration with (a) carrier solution as a mock-treatment, (b) 5 µM FB1, or (c) 100 nM PSK + 5 µM FB1. The same leaves had their length measured 7 days after treatment. Leaf length was measured as the distance from the base of the leaf (at the petiole end) along the midrib to the tip of the leaf. The measurement at 0 day was made before infiltration with treatment solution. Box plots in each graph not sharing the same letter are significantly different (*t*-tests; n =10, $p \leq 0.05$). Box plot horizontal lines indicate the median, and whiskers define the range; outlier data points are shown. While FB1-treated leaves shrunk, mock- and PSK+FB1-treated leaves expanded during the 7-day period.

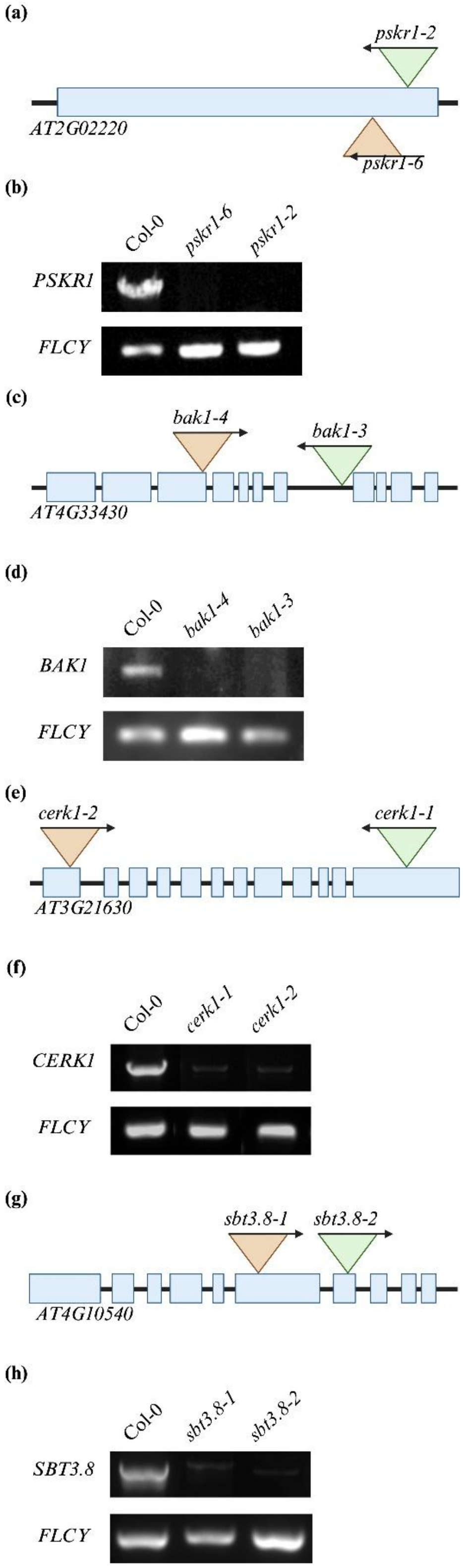

Figure S2. Genotypes of *pskr1*, *bak1*, *cerk1,* and *sbt3.8* mutants. Gene models of (a) *PSKR1*, (b) *BAK1*, (c) *CERK1,* and (d) *SBT3.8* showing the location of T-DNA insertion sites. T-DNA insertion sites are indicated by coloured triangles, with the direction of arrows showing the 5'→3' orientation of the inserts. (e, f, g, h) Genomic DNA was amplified using primers spanning the T-DNA insertion sites. Amplification of the unrelated gene *FLCY* (AT5G63910) from the same DNA samples was used as a reference control.

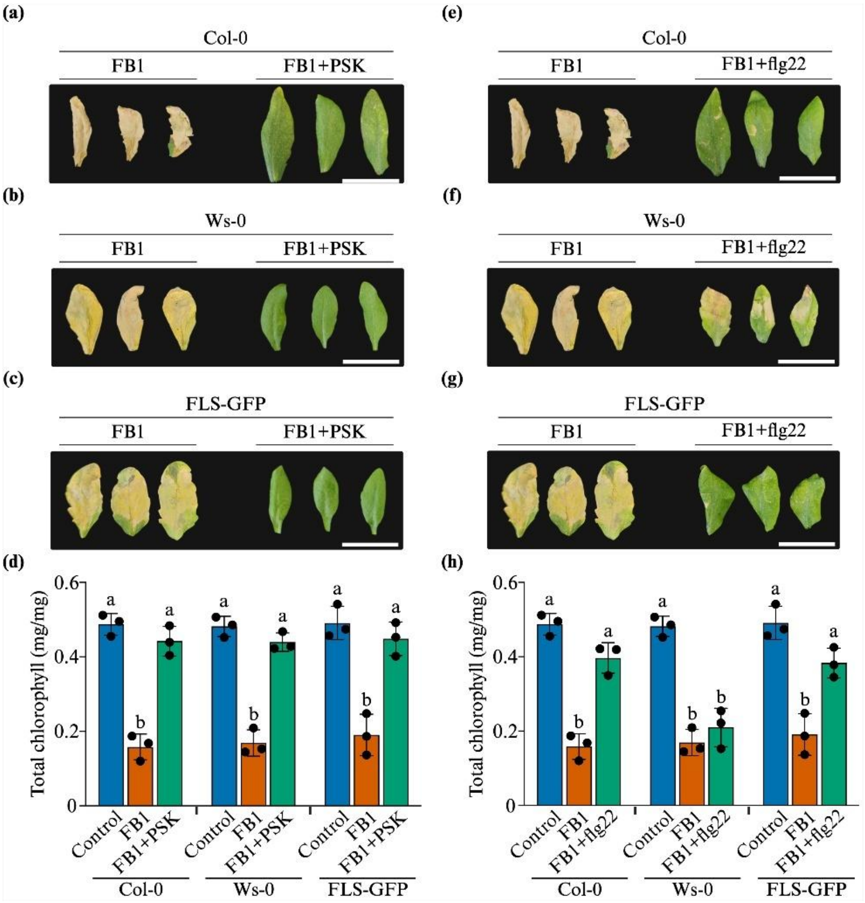

Figure S3. Effects of FLS2 on the cell death-inhibitory functions of PSK and flg22.

Plants of the indicated genotypes were mock-treated or infiltrated with solution consisting of 5 μM FB1 or 100 nM PSK+ 5 μM FB1. After 7 days from treatment, representative leaves were excised for imaging from (a) wildtype Col-0 plants, (b) wildtype Ws-0 plants carrying a naturally occurring *fls2* mutation, and (c) transgenic Ws-0 plants transformed with a gene construct for expression of wildtype FLS2 fused to GFP (FLS2-GFP). (d) Chlorophyll was extracted from similarly treated leaves and quantified 7 days after treatment. (e) Col-0 leaves treated with 5 μM FB1 or 100 nM flg22 + 5 μM FB1, (f) wildtype Ws-0 leaves similarly treated, or (g) transgenic Ws-0 plants expressing the FLS2-GFP construct were photographed after 7 days from treatment. (h) Chlorophyll quantification from leaves treated as indicated for 7 days. Scale bar = 2 cm. Bars represent mean ± SD (n = 3). Statistical analysis was performed using 2-way ANOVA. Bars with different letters are significantly different ($p \leqslant 0.05$). Note that the same FB1-treated leaves of each genotype are used for comparison with FB1+PSK or FB1+flg22 for each genotype.

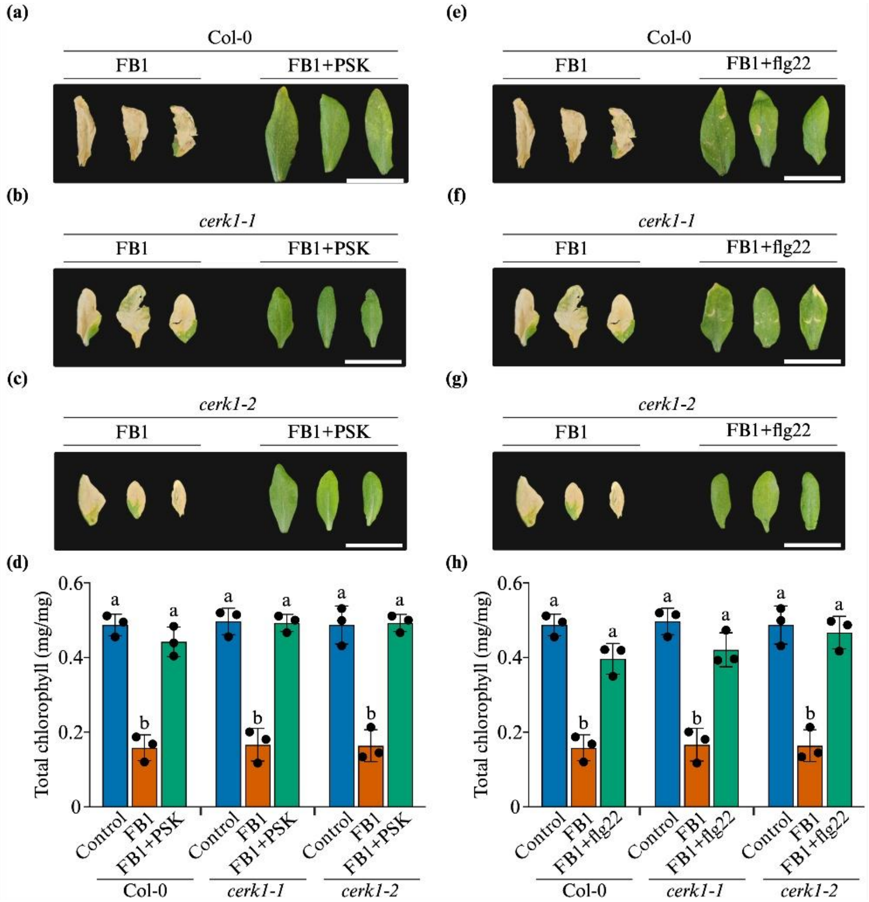

Figure S4. CERK1 is not required for the PSK and flg22 function in blocking FB1 toxicity. Plants of the indicated genotypes were mock-treated or infiltrated with solution consisting of 5 μM FB1 or 100 nM PSK+ 5 μM FB1. After 7 days from treatment, representative leaves were excised for imaging from (a) wildtype Col-0 plants, (b) *cerk1-1* mutant plants, and (c) *cerk1-2* plants. (d) Chlorophyll was extracted from similarly treated leaves and quantified 7 days after treatment. (e) Col-0 leaves treated with 5 μM FB1 or 100 nM flg22 + 5 μM FB1, (f) *cerk1-1* leaves similarly treated, or (g) *cerk1-2* plants were photographed after 7 days from treatment. (h) Chlorophyll quantification from leaves treated as indicated for 7 days. Scale bar = 2 cm. Bars represent mean ± SD (n = 3). Statistical analysis was performed using 2-way ANOVA. Bars with different letters are significantly different ($p \leqslant 0.05$). Note that the same FB1-treated leaves of each genotype are used for comparison with FB1+PSK or FB1+flg22 for each genotype.

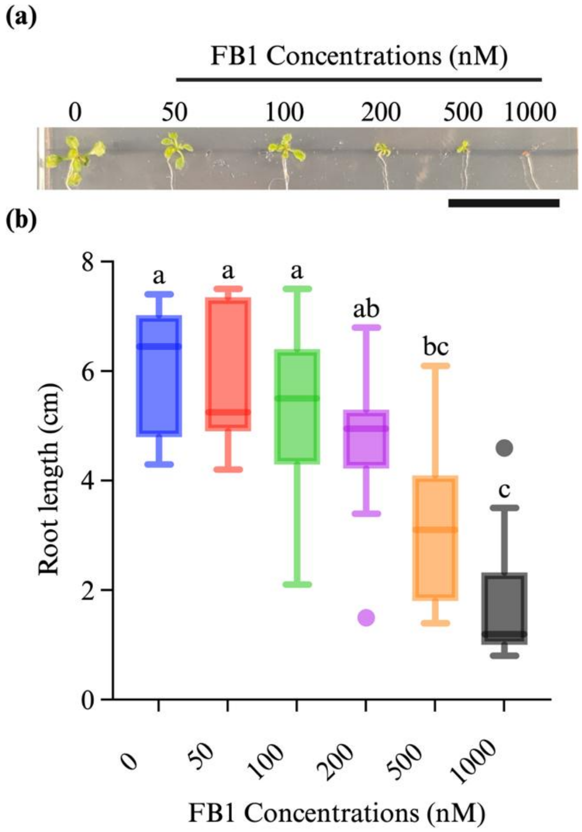

Figure S5. Dose-response effects of FB1 on Arabidopsis growth. (a) Surface-sterilized Arabidopsis seeds were sown on agar plates containing the indicated concentration of FB1. Photographs of the plants to show the appearance of the rosettes were taken after 15 days. Scale bar = 2 cm. (b) Root lengths of similarly treated plants grown on vertical agar plates were measured and plotted against the FB1 concentration. Box-and-whisker plot data were derived from a minimum of 12 replicates per treatment. Different letters above the boxes indicate statistically significant differences between concentrations (ANOVA, Tukey's test, $p \leq 0.05$). Box plot horizontal lines indicate the median, and whiskers define the range; outlier data points are shown.

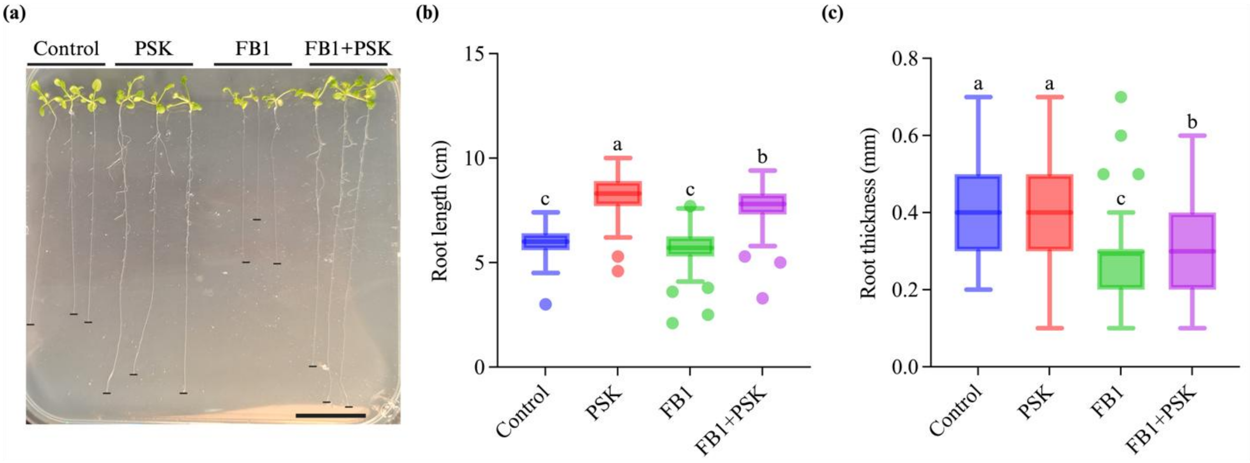

Figure S6. Effects of FB1 and PSK on Arabidopsis growth. (a) Surface-sterilized Arabidopsis seeds were sown on vertical agar plates containing 100 nM PSK, 100 nM FB1, or FB1+PSK at the same concentrations. Representative triplicate plants from each treatment were transferred to a single plate for photography. Scale bar represents 2 cm. (b) A plot of root lengths of plants similarly grown and treated. (c) A plot of root thickness of the same plants in panel b. Box-and-whisker plot data were derived from a minimum of 60 replicate plants. Different letters above the boxes indicate statistically significant differences between concentrations (ANOVA, Tukey's test, $p \leq 0.05$). Box plot horizontal lines indicate the median, and whiskers define the range; outlier data points are shown.

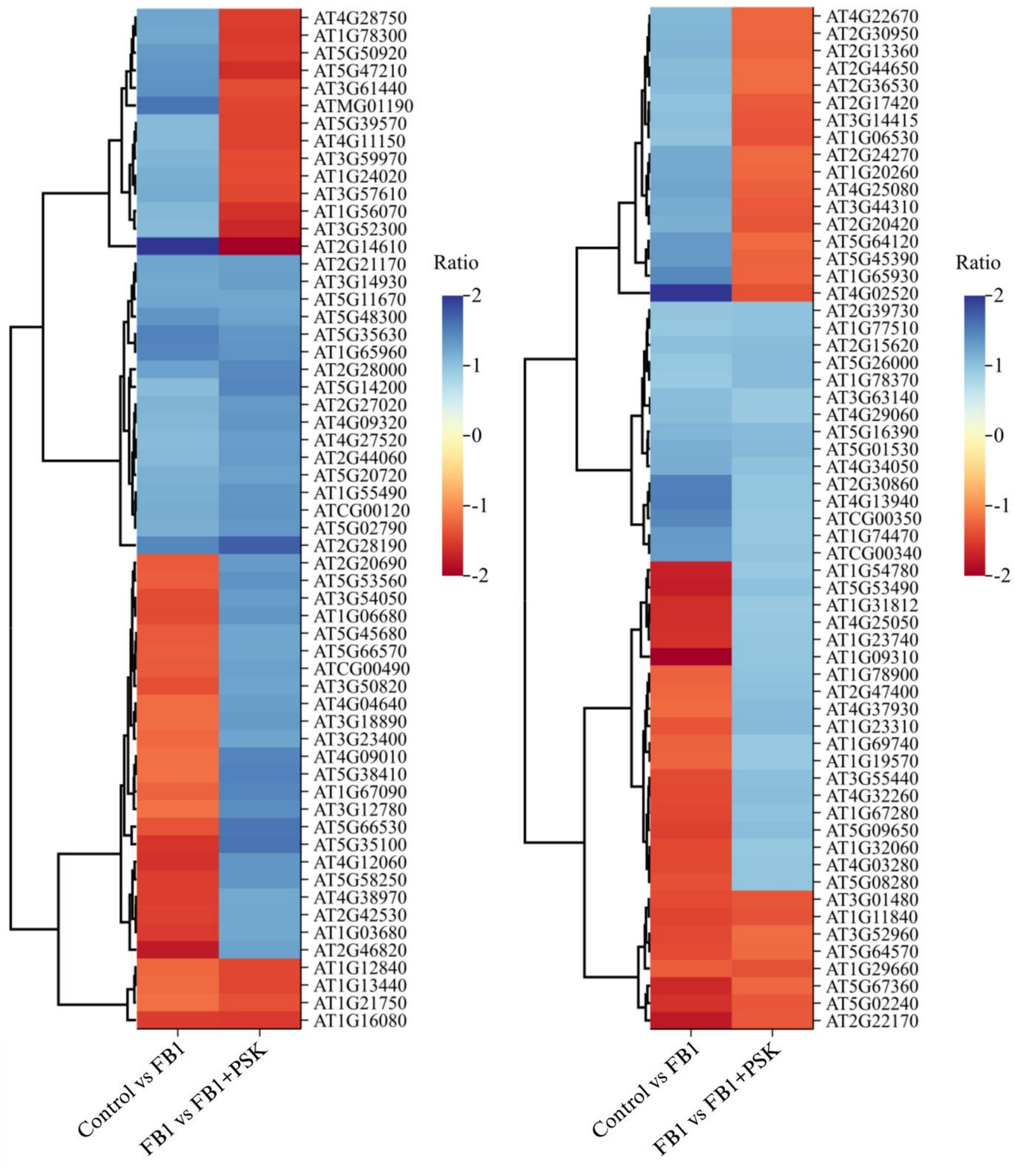

Figure S7. Heatmap view of Arabidopsis proteome response to FB1 and PSK treatments. Arabidopsis seeds were germinated on agar plates with control, 100 nM FB1, or 100 nM PSK + 100 nM FB1 final concentrations. After 15 days, the plants were harvested and protein extracted for iTRAQ analysis. Heatmap shows differentially expressed proteins ($p \leq 0.05$) for the indicated comparisons.

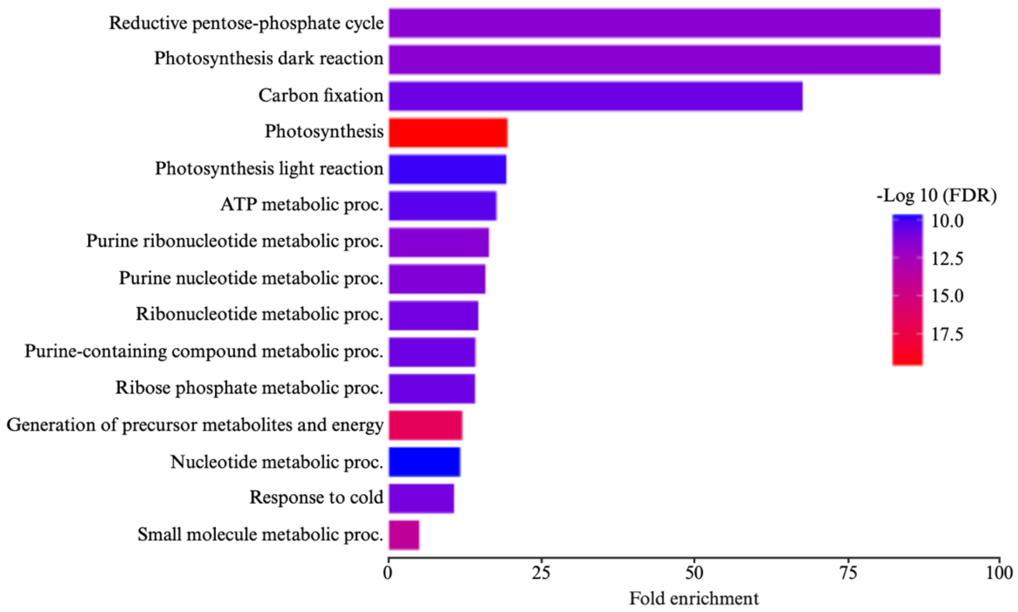

Figure S8. Biological process Gene Ontology enrichment analysis. The enriched biological processes were identified using ShinyGO version 0.82 (Ge *et al*., 2020) from the combined list of proteins responsive to FB1 ± PSK treatments. *P*-values were calculated with the hypergeometric test and corrected for multiple testing using the Benjamini-Hochberg method. Fold enrichment represents the ratio of observed to expected genes in each pathway. The default background (all protein-coding genes) was used. The top 15 enriched biological processes were selected based on FDR (false discovery rate, the expected proportion of false positives among significant results) and fold enrichment.
